# Unknotting RNA: A method to resolve computational artifacts

**DOI:** 10.1371/journal.pcbi.1012843

**Published:** 2025-03-20

**Authors:** Simón Poblete, Mikolaj Mlynarczyk, Marta Szachniuk

**Affiliations:** 1 Facultadde Ingeniería, Arquitectura y Diseño, Universidad San Sebastián, Santiago, Chile; 2 Centro BASAL Ciencia & Vida, Universidad San Sebastián, Santiago, Chile; 3 Institute of Computing Science, Poznan University of Technology, Poznan, Poland; 4 Institute of Bioorganic Chemistry, Polish Academy of Sciences, Poznan,Poland; Hebrew University of Jerusalem, ISRAEL

## Abstract

RNA 3D structure prediction often encounters entanglements, computational artifacts that complicate structural models, resulting in their exclusion from further studies despite the potentially accurate prediction of regions outside the entanglement. This study presents a protocol aimed at resolving such issues in RNA models while preserving the overall 3D fold and structural integrity. By employing the SPQR coarse-grained model and short Molecular Dynamics simulations, the protocol imposes energy terms that enable selective modifications to disentangle structures without causing significant distortions. The method was validated on 195 entangled RNA models from CASP15 and RNA-Puzzles, successfully resolving over 70% of interlaces and approximately 40% of lassos, with minimal impact on the original geometry but notable improvement in ClashScore. The efficiency of untangling conformations that are unequivocally classified as artifacts is 81%. Certain cases, particularly those involving dense packing of atoms or complex secondary structures, posed challenges that limited the efficiency of the method. In this paper, we present quantitative results from the application of the protocol and discuss examples of both successfully disentangled and unresolved structures. We show a viable approach for refining models previously deemed unsuitable due to topological artifacts.

## Author summary

Most algorithms for 3D RNA structure prediction, including a recent version of AlphaFold, in the pool of solutions return models with entanglements, which turn out to be artifacts of computational modeling. Excluding the entanglement region, these models can reflect the native conformation quite well; however, due to the presence of the artifact, they are not considered promising and are therefore discarded from further study. In this paper, we present the first method that enables the systematic classification and removal of RNA entanglements while preserving the model’s consistency and its global 3D fold. Tests on the *in silico* models submitted to RNA-Puzzles and CASP15 RNA show that the system can automatically solve a significant number of issues, including 81% of entanglements considered incorrect conformations.

## Introduction

In recent years, computational modeling has emerged as a leading technique for elucidating the secondary and tertiary structures of biological molecules. Deep learning-driven modeling has already successfully replaced experimental methods in protein research [1–3]. For nucleic acids, predictive algorithms complement wet-lab experiments. Current computational tools effectively generate secondary structures of RNA and DNA from sequences, primarily reflecting short-range canonical interactions. However, non-canonical pairings and long-range contacts are often absent in output models [4–6]. Obtaining the three-dimensional structure of nucleic acids from sequences or base pairing information remains a challenge, as evidenced by genomic studies and blind prediction initiatives such as CASP and RNA-Puzzles [7–11]. Nonetheless, computer-based 2D and 3D structure prediction methods are frequently employed to create initial models of nucleic acid molecules, which are then refined and validated experimentally. *In silico* generated models sometimes verify experimental data and often serve as starting points for designing new RNA- or DNA-based diagnostics and therapeutics, as well as applications in bio- and nanotechnology [12–18]. Thus, the accuracy of structural modeling and the quality of nucleic acid predictions are becoming increasingly important.

Prediction accuracy is typically evaluated in the context of a reference structure, meaning a molecule with a known experimental structure is used as a benchmark. The computer-predicted model is then compared to this reference structure, and similarity or distance measures are calculated. These metrics consider both the 3D structure parameters and the compatibility of secondary interactions; exemplary approaches applied for RNA, the focus of this paper, have been proposed in [19–23]. In contrast, the quality of a computational model can be assessed without referencing a specific target. This involves analyzing the geometry of the structure to ensure that its parameters (such as angles, bond lengths, interatomic distances, and planarity of bases) fall within acceptable ranges [24]. Programs like MAXIT [25] and MolProbity [26] facilitate this process by identifying and often correcting geometric abnormalities in the RNA 3D models. Furthermore, recent tools – KymoKnot [27], Topoly [28], and RNAspider [29] – analyze the topology of 3D structures, identifying topological anomalies such as interpenetrating fragments that form entanglements or complex topological knots, which are unlikely to occur in natural molecules.

Recent studies have shown that all current methods for predicting 3D RNA structures tend to generate entangled models [30, 31]. In response, we present the first systematic solution to this problem, combining the RNAspider tool for entanglement identification with SPQR (SPlit–and–conQueR), a coarse-grained model specifically designed for the prediction and refinement of RNA structure [32, 33]. SPQR can effectively resolve unwanted structural entanglements by allowing the introduction of arbitrary energy terms. These terms can distort the geometry of selected nucleotides while preserving essential RNA interactions and conformations [33–35]. Furthermore, SPQR facilitates the straightforward incorporation of all-atom details [36]. By leveraging data from RNAspider, we can define precise repulsive or attractive interactions between specific nucleotides, minimizing entanglements with marginal impact on the overall geometry of the structure. However, we have identified instances where the disentanglement process cannot be applied without significantly distorting the secondary structure, or where the compactness of the molecule poses challenges to the correction procedure. In the paper, we present the untangling procedure and its results on example RNA 3D models. We also address cases of problematic entanglements in which the method proved ineffective and suggest directions for future research on resolving these challenges in RNA structure prediction and refinement.

**Fig 1:**
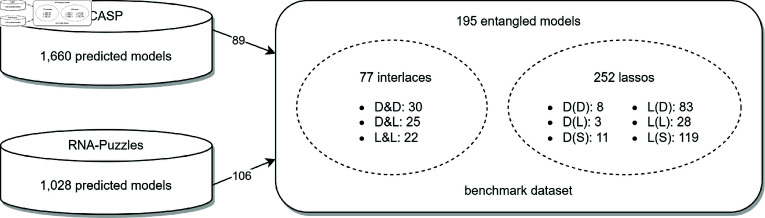
Benchmark data.

## Materials and methods

### Testing dataset

To test the disentanglement protocol, we downloaded RNA 3D models predicted in the CASP15 and RNA-Puzzles competitions, available in their online repositories as of January 2024. The CASP15 dataset (https://predictioncenter.org/download_area/CASP15/predictions/RNA/) included 1,660 models generated in CASP15 for 12 RNA targets. The RNA-Puzzles dataset (https://github.com/RNA-Puzzles) contained 1,028 models targeting 22 RNA sequences in rounds I-IV of RNA-Puzzles. From this collection, we discarded redundant structures and blobs, focusing our analysis on the remaining models for the entanglements. Specifically, among the 122 entangled predictions from CASP15, 21 were redundant, 8 were counted as blobs, and 4 were discarded due to clear artifacts in their coordinates. Blobs generally had a ClashScore greater than 400, making their structures impossible to analyze. The exception was the R1126TS470_5 model, which exhibited an analyzable structure and appeared visibly reasonable despite the high number of clashes and its ClashScore of 416.7. In the remaining set of 89 CASP15 RNA models, 88 had up to 7 entanglements (with 51 of them having only a single entanglement) (see Fig A in S1 Text). One outlier, the R1138TS239_5 model (720 nts), had a ClashScore of 86.1 and contained as many as 15 entanglements. For reference, based on our experience from CASP and RNA-Puzzles, a ClashScore is insignificant and considered acceptable if it is below 10. The RNA-Puzzles collection included 123 entangled structures, 12 of which were discarded as duplicates. Five additional structures were removed due to entanglement resulting from misplaced phosphates. Among the remaining 106 non-redundant models, each contained up to five entanglements, with the vast majority (88 models) exhibiting a single entanglement. The complete distribution of the number of entanglements in each dataset is shown in Fig A in S1 Text. Ultimately, 195 entangled structures were included in the test collection: 89 from CASP15 and 106 from RNA-Puzzles (Fig 1). They contained a total of 329 structural element entanglements classified into 9 groups: 3 classes of interlaces (dinucleotide step-dinucleotide step, labeled D&D; dinucleotide step-loop, labeled D&L; and loop-loop, labeled L&L), 3 classes where a dinucleotide step forms a lasso (around another dinucleotide step, labeled D(D); around a loop, labeled D(L); or around a single strand, labeled D(S)), and 3 classes where a loop forms a lasso (around a dinucleotide step, labeled L(D); around another loop, labeled L(L); or around a single strand, labeled L(S)). Note that in the class labels, D stands for a dinucleotide step, L for a loop, and S for a single strand, & denotes interlace, and brackets are used to denote lasso-type entanglement. A list of models, along with the entanglement information, is provided in S1 Table (CASP15 subset) and S2 Table (RNA-Puzzles subset).

### Identification of entanglements

**Fig 2:**
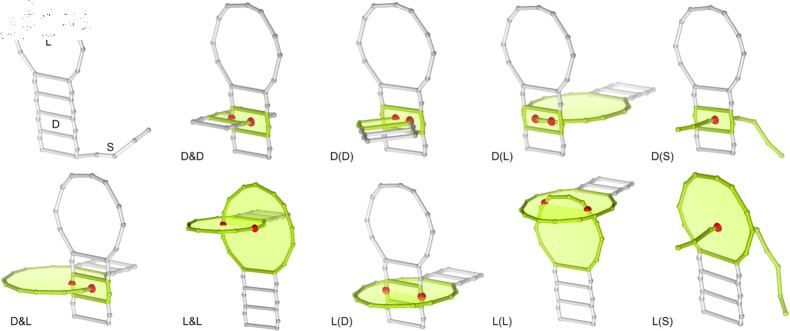
Types of entanglements of structure elements. Elements include loops (L), dinucleotide steps (D), or single strands (S). Intersection points are depicted as red balls.

To identify entanglements of structural elements, we used the RNAspider program with its default settings [29]. RNAspider analyzes the input 3D structure for entanglements, outputs the identified entanglements, and provides their locations. The system can distinguish between three types of interlaces (also called links; D&D, D&L, L&L) and six types of lasso entanglements (D(D), D(L), D(S), L(D), L(L), L(S)) (Fig 2). Each entanglement involves two structural elements that are either circular (loops and dinucleotide steps) or linear (single strands). An interlace is formed by two circular elements, while a lasso-type entanglement involves a circular component that encircles another structural element, which can be of any type. In [31], we further distinguish between shallow and deep L(*) type lassos. Shallow lassos, occurring when the lassoed fragment threaded through the loop spans no more than five nucleotides, are considered non-pathological due to their potential for spontaneous disentanglement. In contrast, deep L(*) lassos and all D(*) type lassos are classified as artifacts.

RNAspider processes the tertiary RNA structure by first applying the RNApolis Annotator [37, 38] to annotate base pairs. This step allows the system to derive the secondary structure of RNA. Based on the latter, RNAspider identifies structural elements within the input 3D structure, such as loops, dinucleotide steps, and single-stranded fragments. Next, the full-atom 3D structure is converted into a wireframe model. In this model, nodes represent selected backbone atoms (P, C4^′^) and the centroids of heavy atoms that form Watson-Crick-Franklin hydrogen bonds, while edges illustrate the RNA backbone and base pairings. Thus, each loop and each dinucleotide step is encircled by a closed polynomial chain. The interior of each circular structure element is covered with a mesh and is recursively triangulated. The subdivision continues until sufficiently small triangles are obtained, with the user able to set a stop criterion. The punctures are checked for each triangle using the Moeller-Trumbore algorithm [39]. This process allows RNAspider to identify intersection points where one structural element penetrates another. Once an intersection point is identified, the system determines which structure elements form the entanglement and whether it is an interlace or a lasso.

### Removal of entanglements

**Fig 3:**
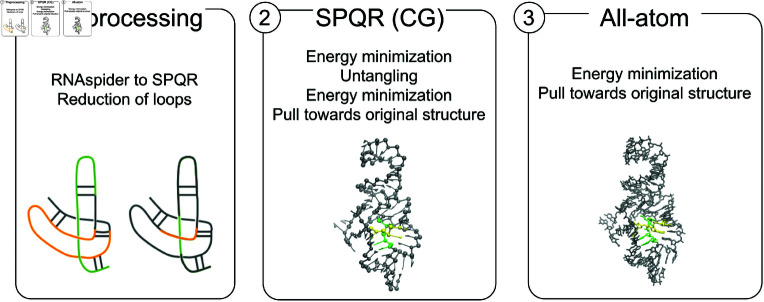
Disentanglement process: (1) RNAspider-to-SPQR data reformatting and reduction of loops around intersection points; (2) SPQR simulations that pull apart the fragments involved in the entanglement; (3) All-atom refinement.

To resolve entanglements of structural elements, we applied the untangling protocol (see Appendix A in S1 Text). The protocol involves a series of simulations utilizing both coarse-grained (CG) and atomistic representations [36], and follows three main steps (Fig 3).

1. Data preprocessing. The output of RNAspider is reformatted for subsequent coarse-grained simulations. First, we process the list of entanglements by identifying pairs that share a common structural element. For each such pair, we examine the remaining structural elements involved in both entanglements to determine if they are somehow connected – for instance, if they are adjacent or if one contains the other. When this condition is met, the two entanglements are merged and treated as a single entanglement for further processing. This step is repeated for all entanglements, resulting in a refined list of disjoint cases, where no two entanglements share a structural element. After this, for each resulting entanglement, single strands and hairpins larger than 5 nucleotides are reduced. This process is applied to L&L and D&L interlaces, as well as L(S) and D(S) lassos. Reduction aims to focus on fragments that are crucial for disentanglement and make the process more efficient. The loops are reduced to smaller segments, retaining the nucleotides that are closest to the intersection points and do not form base pairs. In case that all the nucleotides of the reduced loop are paired, the script looks for a segment where two consecutive bases do not belong to the same dinucleotide step. Although this reduction facilitates more effective untangling simulations, it is not always feasible due to specific secondary structures or the geometry of the entanglement.
2. Coarse-grained simulations. The coarse-grained simulations utilize the SPQR model [32, 33], which strikes a good balance between accuracy and computational efficiency. This model also supports the introduction of virtual sites, which have been successfully used in the removal of simpler links [33]. In the CG simulation stage, initial energy minimization is first employed to eliminate steric clashes and reconstruct any broken bonds. Following this, virtual sites and specific energy terms between them are defined to carefully pull apart the entangled fragments. In addition, the excluded volume interaction between the nucleotides of two entangled loops is turned off. The number of virtual sites depends on the loop type (Fig B in S1 Text). For hairpins, duplexes, internal loops, and junctions, the virtual sites are situated at the center of mass and at the midpoints of the closing base pairs. During the simulation, the repulsive energy term separates the loops, preventing them from crossing over the closing pairs. If the loops have been reduced to shorter strands, as described in the preprocessing step, a simpler and faster method is used. Given two loops, *L_1_* and *L_2_*, we calculate their midpoints and place a fixed virtual site at each. Every loop is repelled by its virtual site and attracted to the opposite one (Fig B in S1 Text). This effectively swaps the positions of the short strands, providing a straightforward way to disentangle them. The functional form of the energy terms involved is described in Appendix B in S1 Text. The untangling simulation proceeds with another brief energy minimization, followed by a short steered Monte Carlo run. This run guides the nucleotides to restore the original shape of the model after untangling its topology. The simulations allow the movement of all particles, providing sufficient flexibility for disentangling the selected structure elements. Distortion in regions far from the entanglement is minimal, as the final steps of energy minimization and the steered run restore their original geometry.
3. All-atom refinement. The atomistic phase is conducted through steered Molecular Dynamics (MD) simulations, which allow for the introduction of fine-grained details into a more realistic structural model. Starting from the coarse-grained model, an atomistic template is applied to each nucleobase, and the energy of the atoms along the backbone is minimized using Amber force field parameters [40]. Finally, energy minimization is carried out in a vacuum using Gromacs [41] with the full Amber force field [40]. During this process, nucleotides are restrained using RMSD and ERMSD restraints, as implemented in the PLUMED package [42].

Once the structures were treated by the disentanglement protocol, their topology was analyzed again with RNAspider. We also examined the secondary structure of the entangled loops after the refinement, since the rearrangement of the structure can produce the disruption of their constitutive base pairs which might turn an entanglement undetectable. For the cases where this happened, we checked visually the presence or absence of the entanglement, and we reported them accordingly in the Supporting Information (S1 Table for CASP15 and S2 Table for RNA-Puzzles).

## Results and discussion

We applied the disentanglement protocol to each of the 195 entangled structures from the benchmark set (the resulting structures are available at doi: 10.5281/zenodo.13840004). Table 1 presents the aggregate results of this experiment. The protocol successfully resolved approximately half (49%) of the entanglements in eligible RNA structures, with 72% of successful cases coming from CASP15 predictions and 28% from RNA-Puzzles. It was notably more effective for interlaces, resolving 77% of cases, compared to 40% for lassos across both datasets. However, it is important to note that lassos are more prevalent – occurring twice as often as interlaces in the CASP15 dataset and 13 times more frequently in the RNA-Puzzles dataset – generally harder to remove and, importantly, may not be artifacts. Artifacts definitively include all types of interlaces and D(*) lassos. Among the 99 such conformations identified in the dataset, 80 (81%) were successfully untangled.

When analyzing results at the whole-structure level – acknowledging that individual RNA structures may contain multiple entanglements – 41% of problematic structures were completely resolved (i.e., all entanglements were removed), 14% were partially resolved (some entanglements were addressed while others remained), and 45% of structures remained unresolved. Among the unresolved cases (87 RNA models), 98% contained lasso-type entanglements, while only 2% involved solely interlaces.

**Table 1:** Statistics on entanglements in the analyzed datasets.

	CASP15	RNA-Puzzles	Total
RNA structures processed	1,660	1,028	2,688
Entangled structures	122	123	245
(A) eligible to disentangle (benchmark set)	89	106	195
- successfully disentangled	45	35	80
- partially disentangled	17	11	28
- failed to disentangle	27	60	87
(B) non-eligible structures (blobs & repeats)	33	17	50
Entanglements in eligible structures	191	138	329
- interlaces	67	10	77
- lassos	124	128	252
(C) successfully disentangled	116	45	161
- interlaces	51	8	59
- lassos	65	37	102
(D) failed to disentangle	75	93	168
- interlaces	16	2	18
- lassos	59	91	150

We evaluated the impact of disentangling on overall structure quality by analyzing changes in RMSD (Root Mean Square Deviation) [19], INF (Interaction Network Fidelity) [20], and ClashScore [43] after applying the protocol. S3 Table (for CASP15) and S4 Table (for RNAPuzzles) provide detailed values of these measures for all models in the benchmark set, while Table 2 summarizes the results of their analysis.

**Table 2:** Statistics on evaluation measures for RNA models with at least one resolved entanglement (108 cases).

	ClashScore	RMSD	INF
#(%) structures with better rating	70 (65%)	26 (24%)	14 (13%)
#(%) structures with worse rating	37 (34%)	80 (74%)	76 (70%)
#(%) structures with unchanged rating	1 (1%)	2 (2%)	18 (17%)
Average improvement ( ± SD)	88.74 ± 105.65	0.5 ± 0.7	0.02 ± 0.01
Average deterioration ( ± SD)	7.38 ± 6.56	0.3 ± 0.7	0.04 ± 0.03

RMSD (distance measure) and INF (similarity measure) were calculated relative to the reference structure both before and after disentanglement. When multiple reference models were available (as in some CASP15 targets), the first model was used. ClashScore, a reference-free measure of stereochemical quality, did not require comparison to the native structure. It showed significant improvement after refinement – 70 out of 108 RNA models had a lower post-refinement ClashScore (the largest decrease was 410.97), especially in larger RNA models. For 37 out of 108 models, the measure increased slightly (the largest increase was 25.57), mostly in cases where the pre-refinement ClashScore was already low, and the structures were densely packed in 3D space. Changes in RMSD and INF values were negligible, confirming that the untangling protocol preserves conformational integrity. RMSD differences ranged from *–*3*.*5Å to 5.9Å, while INF were between -0.12 and 0.04. The small reduction in INF was mostly attributed to the disentanglement of loops, which were stabilized by base pairs. Discrepancies in these values can be improved by running simulations with stronger restraints in the last steps of the refinement procedure, for which we report a range of reasonable values in Appendix A in S1 Text.

We also tested the disentanglement protocol on selected blobs and found them generally difficult to relax during Step 1 of the procedure. Additionally, Step 2 presented challenges in disentangling due to insufficient space to accommodate the loops in the resulting structure. Consequently, we confirmed that the blobs were not suitable for untangling.

Finally, we assessed the time required for disentanglement. These tests were conducted on two machines with different configurations: MacOS 12.6 (i5-8259U, 4 cores/8 threads) and Linux Ubuntu 20.04.1 (AMD Ryzen Threadripper 2950X, 16 cores/32 threads). For a medium-sized structure (363 nucleotides, 1 entanglement), the disentanglement simulations took approximately 2 minutes, while the backmapping relaxation step required an additional 5 minutes. The runtime for disentanglement varied based on the structure size and the number of entanglements, ranging from a few seconds for smaller structures (<100 nucleotides) to several minutes for larger ones (>700 nucleotides). The backmapping relaxation time depended solely on the structure size, ranging from 1 to 15 minutes. Lastly, the all-atom refinement step, performed with Gromacs 2021.2 using 8 threads and a single GPU, required 4 minutes for a 69-nucleotide structure and up to 12 minutes for a 720-nucleotide structure.

**Fig 4:**
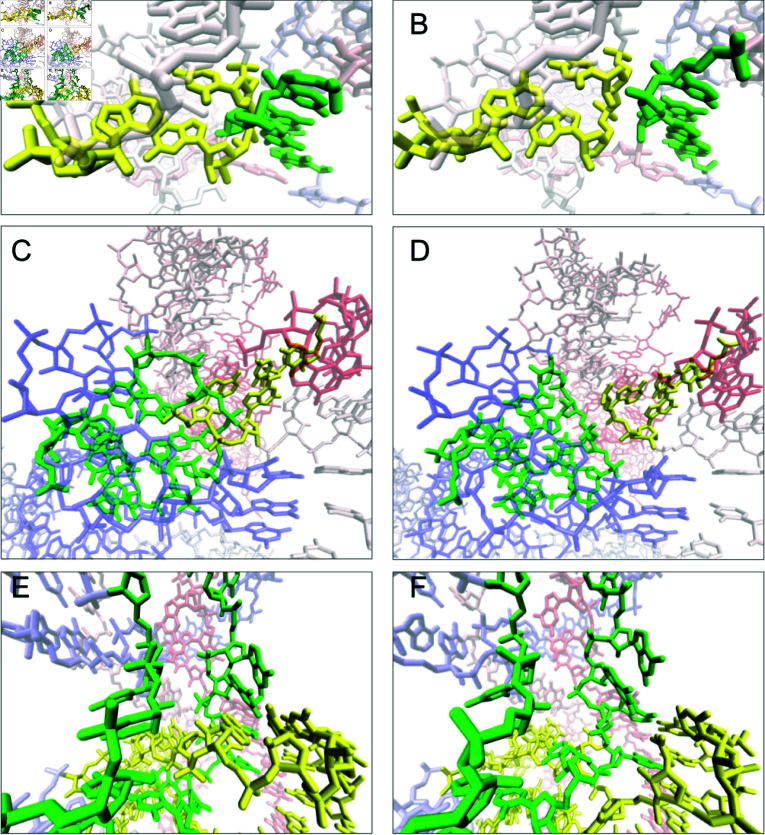
Example interlaces in the dataset: D&D in R1126TS110_1 – (A) linked and (B) resolved; D&L in PZ28_Bujnicki_4 – (C) linked and (D) resolved; L&L in PZ24_Kollmann_3 – (E) reduced and linked, (F) reduced and resolved.

### Interlaces

Interlaces typically arise from the superposition of fragments, leading to entangled geometry after eliminating the clashes. These include D&D, D&L, and L&D conformations, all of which are considered artifacts. Fig 4 presents examples of successfully resolved interlaces for each type.

Our protocol effectively disentangles D&D interlaces, achieving a high success rate. Of the 30 D&D entanglements in RNA models from the benchmark set, 29 were successfully resolved (97%). In the single unresolved case, two large fragments of the structure overlapped, spanning six loops and creating five adjacent entanglements (Fig C in S1 Text). The attempt to simultaneously remove all these entanglements was unsuccessful.

Similarly, for D&L entanglements, the disentanglement protocol proved highly efficient, resolving 18 out of 25 cases (72%). The permanent interlaces of this type are distributed across five RNA structures. In three of these (CASP15 models), the D&L interlaces coexist with at least four other entanglements. In the fourth unresolved structure (also from CASP15), numerous clashes indicated by a high ClashScore, lead to a compact geometry that leaves no space for proper disentanglement (cf S1 Table and S3 Table). In the fifth unresolved model, from RNA-Puzzles, the disentanglement protocol transformed the D&L interlace into an L(D) lasso.

**Fig 5:**
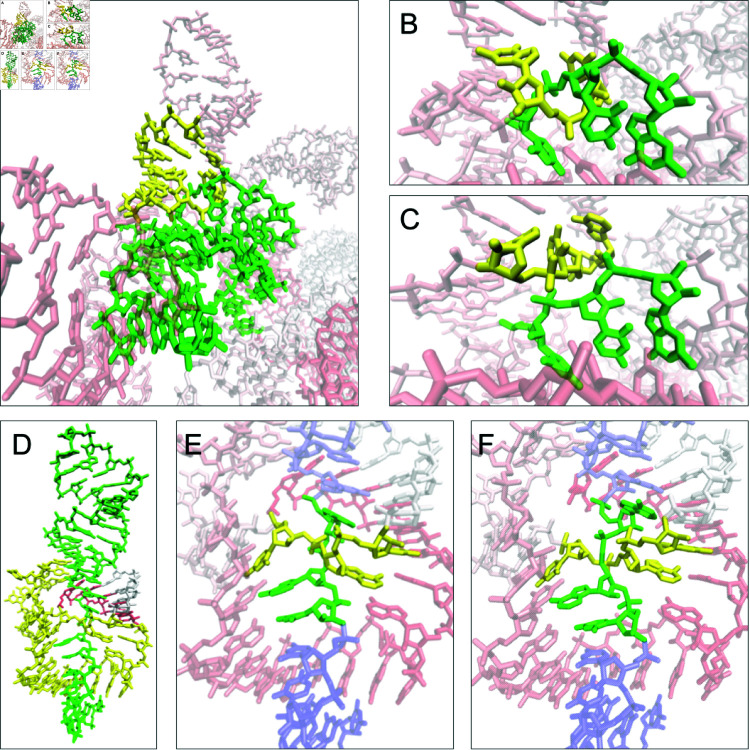
Reduction and removal of the R1138TS076_3 interlace L&L, (A) unreduced, (B) reduced and (C) reduced and untangled. For L(S) lasso from the R1107TS054_3 model: (D) unreduced, (E) reduced, (F) reduced and untangled.

For L&L interlaces, the disentanglement protocol is less efficient, successfully resolving 12 out of 22 cases (55%). Failures typically occur when the interlaces are adjacent to other entanglements or when the puncturing loop cannot be reduced to a shorter segment during preprocessing. When loops remain extended, the virtual sites used in refinement simulations become less localized around the entanglement, reducing the procedure’s effectiveness. The reduction of the loops is crucial for an efficient untangling simulations, and it can be significant as shown in Fig 5 for both lassos and interlaces. In both datasets, CASP15 and RNA-Puzzles, we observed instances where L&L interlaces degenerated into L(L) lassos. This outcome can be attributed to the specific geometry, where reducing the loops around the intersection point is not optimal. In such cases, the disentangling simulation displaces the loops in the wrong direction (Fig D in S1 Text). To prevent this, manual intervention in loop reduction, disregarding the intersection point, can be employed.

### Lassos

Lassos include the following categories of entanglements: D(D), D(L), D(S), L(D), L(L), and L(S), and they require treatment different from that of interlaces. In principle, L(D), L(L), D(D), and D(L) can be resolved through a relaxation procedure that fixes atomic clashes. However, they may reappear during the final step of disentanglement simulations when the nucleotides are rearranged to their original conformation. Furthermore, it is essential to recognize that the presence of certain lassos does not necessarily indicate an unphysical situation [44, 45]. For instance, shallow L(*) lassos – those in which the strand fragment passes through the lassoing loop to a depth of no more than 5 nucleotides – can spontaneously disentangle. Consequently, such conformations are not permanent kinetic traps, and we do not classify them as artifacts.

**Fig 6:**
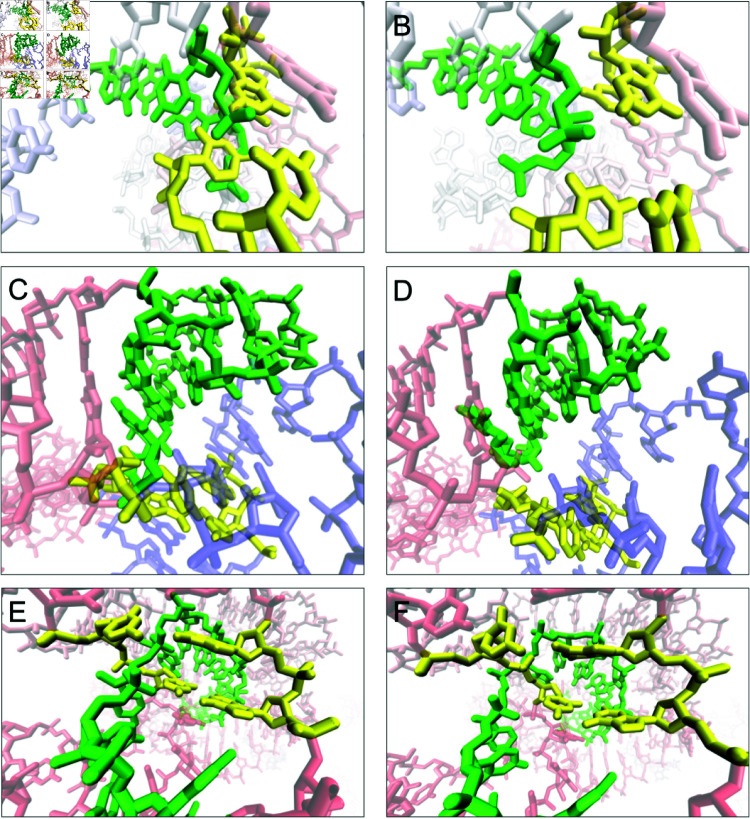
Example lassos in CASP15 models: D(D) in R1116TS470_1 – (A) entangled and (B) resolved; D(L) in R1136TS110_4 – (C) linked and (D) resolved; D(S) in R1138TS185_4 – (E) entangled and (F) resolved.

In the benchmark set, dinucleotide steps occur as lassoing elements 10 times less frequently than loops. This is desirable because D(*) entanglements represent an unnatural conformation that should not appear in RNA structures. A total of 22 D(*) lassos were identified in the analyzed structures, the vast majority (95%) of which were easily removed using the disentanglement protocol. Specifically, the protocol successfully removed all 8 D(D) lassos, all 3 D(L) lassos, and 10 out of 11 D(S) lassos. In the only unresolved case, the lasso coexisted with interlaces involving adjacent fragments of the structure, which complicated the disentanglement process (Fig E in S1 Text). Examples of successfully removed D(*) lassos are illustrated in Fig 6.

L(*) lassos constitute the largest and most problematic category of entanglements, encompassing both artifacts and potentially valid conformations. In the studied dataset, 230 instances of L(*) lassos were identified, of which 102 (44%) were successfully disentangled. Approximately one-third of the unsuccessful cases involved loops closed by a pseudoknot, while the remaining failures were due to various other factors.

The disentanglement of the L(D) and L(L) lassos tends to fail when the lassoing loop forms a large n-way junction big enough to enclose smaller loops, as clearly observed in 5 benchmark structures (see Fig F in S1 Text) . Additionally, the protocol occasionally transforms one type of entanglement into another. For instance, in three RNA-Puzzles models, L(L) lassos were converted into L&L interlaces, as only one fragment of a strand moved far enough to exit the lassoing loop (Fig G in S1 Text).

As for the L(S) lassos, they are quite abundant and exhibit diverse behavior. In 40 cases, the L(S) entanglement is formed by a dangling end that punctures the loop. These include 3 shallow and 37 deep lassos. We identified such instances in 11 models, where a short 3-4 nucleotide tail of the RNA strand penetrated the loop. Although these lassos were often resolved during the untangling process, they frequently reappeared in the final steps of the procedure. Additionally, 36 successfully disentangled structures in the RNA-Puzzles set contained lassos stabilized by base pairs. This situation can be assessed before disentanglement by annotating the structure prior to the refinement procedure and searching for interactions within the entangled loops. As a consequence, refinement may lead to the distortion or destruction of base pairs present in the target structure, potentially reducing the INF value (or increasing the RMSD). An example of this situation is illustrated in Figure H in S1 Text.

An interesting behavior was observed in one RNA-Puzzles model containing an L(S) lasso. During the untangling process, distortion of a single strand led to the formation of a new L(S) lasso, with the loop partially composed of the strand from the original entanglement (Fig I in S1 Text). A similar situation occurred in the other model from the RNA-Puzzles dataset, where the removal of L(S) involving a pseudoknot-closed loop resulted in the creation of a new L&L interlace. This interlace spanned two loops not closed by pseudoknots, one of which shared sections with the single strand from the original entanglement. This example illustrates the complexity of removing lassos when multiple base pairs and pseudoknots are involved.

Lastly, let us mention that the untangling protocol may, in some specific cases, generate new entanglements during the clash removal step. This scenario can occur when the overlap of two loops with significant clashes is not detected as an entanglement by RNAspider but becomes problematic after atomic superposition is gone (Fig J in S1 Text).

## Conclusion

In this work, we have developed a systematic procedure for detecting entanglements and proposing refined untangled structures. Following the identification of entanglements and the specification of core nucleotides to compose them, our pipeline utilizes a multiscale approach that enables rapid energy minimization and manipulation of the nucleotides. The resulting structures are backmapped to a full-atom representation consistent with the original model. The disentanglement protocol was applied to RNA models predicted in CASP15 and RNA-Puzzles and led to the observation of substantial differences in the behavior of interlaces and lassos. For interlaces, which are clearly topological artifacts, the results are generally favorable and indicate that the protocol can be applied reliably in most cases. In contrast, the scenario for lassos is more complex. Untangling lassos often disrupts base pairs stabilizing them, or may have little to no effect on the overall structure of RNA. This indicates that the entanglement may be non-removable or that it poses no significant harm to the integrity of the molecule.

Overall, the refinement significantly improves the ClashScore, particularly for structures with a high number of clashes. However, for clash-free RNAs, molecular dynamics (MD) treatment may inadvertently introduce some clashes. Addressing this issue will be part of future work aimed at establishing a more robust framework. A similar observation applies to the contact maps, as the INF values experience a slight decrease. MD refinement is crucial in this context, and the use of more specific force fields will facilitate better structure stabilization and automated reliability assessment. In summary, future research will focus on further enhancing the method to address the remaining challenges in disentangling RNA structures, ultimately improving the accuracy and utility of computational RNA models for biological studies and biotechnological applications.

## Supporting information

S1 Table Entanglements in the benchmark set (CASP15 predictions). Entanglements are color-coded as follows: black - successfully disentangled, red - unresolved, orange - transformed to another type, blue - generated by the protocol. A star (*) denotes cases where the 2D structure of at least one entangled loop was affected, requiring manual verification of entanglements.
(PDF)

S2 Table Entanglements in the benchmark set (RNA-Puzzles predictions). Entanglements are color-coded as follows: black - successfully disentangled, red - unresolved, orange - transformed to another type, blue - generated by the protocol. A star (*) denotes cases where the 2D structure of at least one entangled loop was affected, requiring manual verification of entanglements.
(PDF)

S3 Table Evaluation of CASP15 predictions before and after applying the disentanglement protocol. *Δ* represents the difference between the post- and pre-disentanglement measurements (*Δ* = After – Before). *n/a* indicates that the protocol failed for the given model, so post-disentanglement and *Δ* values are not available.
(PDF)

S4 Table Evaluation of RNA-Puzzles predictions before and after the application of the disentanglement protocol. *Δ* represents the difference between the post- and pre-disentanglement measurements (*Δ* = After – Before). *n/a* indicates that the protocol failed for the given model, so post-disentanglement and *Δ* values are not available.
(PDF)

S1 Text Contains Figs A–J with their descriptions and Appendices A and B. (PDF)
