# Supplementary material for: Unknotting RNA: A method to resolve computational artifacts
Source: PLoS Comput Biol. 2025 Mar 20;21(3):e1012843. doi: 10.1371/journal.pcbi.1012843 (PMC11925458; doi:10.1371/journal.pcbi.1012843)
Supplement: S1 Table — Entanglements are color-coded as follows: black - successfully disentangled, red - unresolved, orange - transformed to another type, blue - generated by the protocol. A star (*) denotes cases where the 2D structure of at least one entangled loop was affected, requiring manual verification of entanglements. (PDF) [file pcbi.1012843.s001.pdf]

Table S1: Entanglements in the benchmark set (CASP15 predictions): black - successfully disentangled, red - unresolved, orange - transformed to another type, blue - generated by the protocol. A star (*) denotes cases where the 2D structure of at least one entangled loop was affected, requiring manual verification of entanglements.

No	Target	Model	Entanglements
1	1117	TS097_1	L(S)
2	1107	TS054_1	L(S)
3	1107	TS054_3	L(S)
4	1107	TS054_4	L(S)
5	1107	TS054_5	L(S)
6	1107	TS119_3	L(S)
7	1107	TS125_1	L(S)
8	1107	TS125_2	L(S)
9	1107	TS416_2	L(S)
10	1107	TS416_3	L(S)
11	1116	TS029_3	L(S)
12	1116	TS177_1	L(S)
13	1156	TS119_5	L(S)
14	1136	TS054_3	L&L
15	1136	TS054_4	L(L)
16	1136	TS119_2	L(L)
17	1138	TS229_4	L&L
18	1149	TS097_1	L(S)
19	1138	TS185_1	L(S)
20	1138	TS185_4	D(S)
21	1128	TS110_1	D&L*
22	1126	TS470_1	L(D)
23	1126	TS470_2	D(S)
24	1108	TS119_5	L(S)
25	1126	TS035_1	L(L)
26	1126	TS076_2	L&L
27	1128	TS239_3	L(S)
28	1149	TS177_1	L(S)
29	1136	TS029_2	L(L)
30	1136	TS392_1	L(L)
31	1138	TS035_4	D&D*
32	1126	TS110_1	D&D
33	1126	TS110_2	D&D
34	1126	TS110_3	D&D
35	1128	TS416_4	D&D
36	1136	TS110_1	D&D
37	1107	TS392_1	L(S)
38	1107	TS392_2	L(S)
39	1107	TS392_3	L(S)
40	1107	TS392_4	L(S)
41	1107	TS392_5	L(S)
42	1156	TS177_1	L(L)
43	1116	TS248_3	L(S)
44	1126	TS185_1	L(D)*

Continued on next page

Table S1 – continued from previous page

No	Target	Model	Entanglements
45	1126	TS185_2	L(D)*
46	1136	TS470_5	D&L*
47	1128	TS470_3	L(D)
48	1138	TS076_4	D&L
49	1126	TS054_1	L(D)
50	1136	TS147_3	L(D)
51	1138	TS076_3	L&L
52	1156	TS054_5	2×L(S)
53	1126	TS177_1	L(D) L(S)
54	1128	TS285_3	2×D(S)
55	1126	TS470_4	D(L)+L(D) L(D)*
56	1136	TS110_3	L(L) L(D)
57	1116	TS470_5	2×D&D L(D)*
58	1107	TS119_2	L(L) L(D)*
59	1107	TS128_2	L(L) L(D)*
60	1108	TS128_2	L(L) L(D)*
61	1136	TS128_5	2×D&L 2×L(D)
62	1136	TS239_3	L(L) D&L
63	1126	TS147_5	2×L(D)
64	1107	TS163_1	2×L(S)
65	1136	TS470_2	D&D L&L L(S)*
66	1126	TS185_4	D&L L(S)
67	1126	TS128_2	2×L(D)
68	1126	TS416_5	D&D L&L*
69	1156	TS235_5	L(D) 2×L(S)
70	1126	TS239_5	3×D(S)*
71	1116	TS470_3	D&D D&L L(D) L(D)*
72	1116	TS035_3	L&L → L(L) 2×L(D)*
73	1116	TS035_4	L&L → L(L) 2×L(D)*
74	1116	TS035_5	L&L → L(L) 2×L(D)*
75	1116	TS285_4	L(L) 2×L(D)
76	1136	TS128_4	2×D&L L(D)*
77	1126	TS470_5	D(S) 2×L(S) L(S)
78	1128	TS470_5	D(D) L&L D&L L(D)*
79	1128	TS392_3	L(D) 3×L(D)
80	1126	TS185_5	L&L L&L L(D) L(S)*
81	1138	TS054_3	L&L 3×L(D)
82	1138	TS076_2	D&D 2×D&L 2×L(D)*
83	1128	TS238_2	2×D&D L&L D&L L(D)*
84	1116	TS470_1	4×D&D D(D)+D(D) 2×D(D)*
85	1136	TS110_4	2×D&D D&L 2×D(D) D(L)*
86	1126	TS444_1	2×D&D D&L D(S) L(S) 2×L(S)*
87	1136	TS177_1	3×L(D) 4×L(S)*
88	1136	TS110_2	4×D&D 2×D&L L(D)*
89	1138	TS239_5	4×D&D 2×D&L L&L 2×D&L D(D) D(S) 2×L(D) L(L)+L(L) D(L)+L(D)*
