# Supplementary material for: Unknotting RNA: A method to resolve computational artifacts
Source: PLoS Comput Biol. 2025 Mar 20;21(3):e1012843. doi: 10.1371/journal.pcbi.1012843 (PMC11925458; doi:10.1371/journal.pcbi.1012843)
Supplement: S2 Table — Entanglements are color-coded as follows: black - successfully disentangled, red - unresolved, orange - transformed to another type, blue - generated by the protocol. A star (*) denotes cases where the 2D structure of at least one entangled loop was affected, requiring manual verification of entanglements. (PDF) [file pcbi.1012843.s002.pdf]

Table S2: Entanglements in the benchmark set (RNA-Puzzles predictions): black - successfully disentangled, red - unresolved, orange - transformed to another type, blue - generated by the protocol. A star (*) denotes cases where the 2D structure of at least one entangled loop was affected, requiring manual verification of entanglements.

No	Target	Model	Entanglements
1	PZ16b	3dRNAAS2_3	D(S)*
2	PZ18	Chen_1	L(S)
3	PZ18	Chen_2	L(S)
4	PZ18	Das_1	L(S)
5	PZ18	Das_2	L(S)
6	PZ18	Das_3	L(S)
7	PZ18	Das_4	L(S)
8	PZ18	DasORIGINAL_2	L(S)
9	PZ18	DasORIGINAL_3	L(S)
10	PZ18	Dokholyan_1	L(S)
11	PZ18	Dokholyan_2	L(S)
12	PZ18	RNAComposer_5	L(D)
13	PZ18	solution_0	L(S)
14	PZ24	DasTFN_2	L(S)
15	PZ24	DasTFN_4	L(S)
16	PZ24	DasTFN_5	L(S)
17	PZ24	FARFAR2_1	L(S)
18	PZ24	FARFAR2_2	L(S) L&L*
19	PZ24	FARFAR2_3	L(S)
20	PZ24	FARFAR2_5	L(S)
21	PZ24	iFoldRNA_4	L(D)*
22	PZ24	Kollmann_3	L&L
23	PZ24	Kollmann_6	L(D) L(L) → L&L*
24	PZ24	Kollmann_8	L(S)
25	PZ24	Vfold3D_3	L(S)
26	PZ25	Das_2	L&L → L(L) L(D) L(S)*
27	PZ25	Ding_1	L(S)
28	PZ25	Ding_2	L(S)
29	PZ25	Ding_3	L(S)*
30	PZ25	SimRNA_3	L(S) L(S)*
31	PZ26	Chen_9	L(S)
32	PZ26	Das_3	L(S)
33	PZ26	FARFAR2_5	L(S)
34	PZ26	TFN_3	L(S)
35	PZ26	TFN_6	L(S)
36	PZ26tBox	Chen_9	L(S)
37	PZ26tBox	Das_3	L(S)
38	PZ26tBox	FARFAR2_5	L(S)
39	PZ26tBox	TFN_3	L(S)
40	PZ26tBox	TFN_6	L(S)
41	PZ27	Das_2	L(S)
42	PZ27	Das_6	L(S)
43	PZ27	FARFAR2_6	L(S)
44	PZ27	RNAComposer_2	L(L)

Continued on next page

Table S2 – continued from previous page

No	Target	Model	Entanglements
45	PZ27	SimRNA_3	L(L) → L&L L(D)
46	PZ27tBox	Das_2	L(S)
47	PZ27tBox	Das_6	L(S)
48	PZ27tBox	FARFAR2_6	L(S)
49	PZ27tBox	RNAComposer_2	L(L)
50	PZ27tBox	SimRNA_3	L(L) → L&L L(D)
51	PZ28	Bujnicki_4	L&L D&L L(D)
52	PZ28	Bujnicki_5	L&L
53	PZ28	SimRNA_5	L(D)
54	PZ28tBox	Bujnicki_4	L&L D&L L(D)*
55	PZ28tBox	Bujnicki_5	L&L
56	PZ28tBox	SimRNA_5	L(D)
57	PZ31	Dfold_3	L(S)
58	PZ31	Dfold_4	L(D)*
59	PZ32	Boniecki_1	L(S)
60	PZ32	Boniecki_2	L(S) L(S)
61	PZ32	Boniecki_3	L(S)
62	PZ32	Boniecki_4	L(L)
63	PZ32	Bujnicki_1	2×L(D) 2×L(S)
64	PZ32	Chen_5	2×L(D) L(S)
65	PZ32	Dfold_3	L(L)*
66	PZ32	Dfold_6	L(S)*
67	PZ32	Dfold_10	L(D)*
68	PZ32	FARFAR2_3	2×L(D) 2×L(S)
69	PZ32	FARFAR2_5	L(D)
70	PZ32	FARFAR2_6	2×L(D) 2×L(S)
71	PZ33	Dfold_3	L(L)*
72	PZ33	Dfold_6	L(S)*
73	PZ33	Dfold_10	L(D)*
74	PZ34	Chen_7	L(S)
75	PZ34	Chen_9	L(S)
76	PZ34	Nithin_4	L(D) 3×L(D)*
77	PZ34	SimRNA_1	L(D) 4×L(D)*
78	PZ35	Bujnicki_2	L(L) L(D)
79	PZ35	Bujnicki_5	L(S)
80	PZ35	RNAComposer_2	L(S)
81	PZ35	RNAComposer_3	L(L)
82	PZ35	RNAComposer_4	L(L) L(S)
83	PZ35	RNAComposer_5	L(S)
84	PZ35	TSR04_2	L(L) L(D)
85	PZ35	TSR04_5	L(S)*
86	PZ35	TSR07_2	L(S)
87	PZ35	TSR07_3	L(L)
88	PZ35	TSR07_4	L(L) L(S)
89	PZ35	TSR07_5	L(S)
90	PZ36	Bujnicki_5	L(S)*
91	PZ36	Nithin_5	L(D)
92	PZ36	RNAComposer_3	L(S)

Continued on next page

Table S2 – continued from previous page

No	Target	Model	Entanglements
93	PZ36	RNAComposer_4	L(S)
94	PZ36	RNAComposer_5	L(S)
95	PZ37	DasARESapo_2	L(S)
96	PZ37	DasARESapo_3	L(S)
97	PZ37	DasFARFAR2apo_3	L(S)
98	PZ37	DasFARFAR2ARESholo_1	L(S)
99	PZ37	DasSWMapo_1	L(S)
100	PZ37	Ding_1	L(S)
101	PZ37	Yang_2	L(S)
102	PZ37	Yang_10	L(S)
103	PZ39	Dfold_4	L(D)
104	PZ39	Perez_7	D&L → L(D) L(D)
105	PZ39	Perez_9	L(S)
106	PZ39	Xiao_1	L&L*
