# Supplementary material for: Unknotting RNA: A method to resolve computational artifacts
Source: PLoS Comput Biol. 2025 Mar 20;21(3):e1012843. doi: 10.1371/journal.pcbi.1012843 (PMC11925458; doi:10.1371/journal.pcbi.1012843)
Supplement: S3 Table — Δ represents the difference between the post- and pre-disentanglement measurements (Δ = After – Before). n/a indicates that the protocol failed for the given model, so post-disentanglement and Δ values are not available. (PDF) [file pcbi.1012843.s003.pdf]

Table S 3: Evaluation of CASP15 predictions before and after applying the disentanglement protocol. Δ represents the difference between the post- and pre-disentanglement measurements (Δ = After – Before). *n/a* indicates that the protocol failed for the given model, so post-disentanglement and Δ values are not available.

No	Target	Model	Clashscore			RMSD			INF		
			Before	After	Δ	Before	After	Δ	Before	After	Δ
1	1117	TS097_1	0	n/a	n/a	13.7	n/a	n/a	0.29	n/a	n/a
2	1107	TS054_1	0.45	15.38	14.93	7.7	7.5	-0.2	0.83	0.81	-0.02
3	1107	TS054_3	0	14.95	14.95	8.1	7.8	-0.4	0.78	0.75	-0.03
4	1107	TS054_4	6.78	9.08	2.3	7.5	7.3	-0.2	0.84	0.86	0.02
5	1107	TS054_5	0.45	13.14	12.69	7.8	7.8	0	0.76	0.75	-0.01
6	1107	TS119_3	1.81	n/a	n/a	13.8	n/a	n/a	0.77	n/a	n/a
7	1107	TS125_1	0	11.77	11.77	7.8	7.6	-0.2	0.76	0.76	0
8	1107	TS125_2	0	n/a	n/a	7.8	n/a	n/a	0.76	n/a	n/a
9	1107	TS416_2	9.5	8.15	-1.35	7.8	7.7	-0.2	0.77	0.75	-0.02
10	1107	TS416_3	4.52	n/a	n/a	7.7	n/a	n/a	0.76	n/a	n/a
11	1116	TS029_3	44.31	6.96	-37.35	42.5	42.8	0.3	0.38	0.41	0.03
12	1116	TS177_1	255.59	n/a	n/a	38	n/a	n/a	0.4	n/a	n/a
13	1156	TS119_5	3.23	3.23	0	18.9	18.8	-0.1	0	0	0
14	1136	TS054_3	0	n/a	n/a	38.7	n/a	n/a	0	n/a	n/a
15	1136	TS054_4	5.74	n/a	n/a	28.3	n/a	n/a	0.89	n/a	n/a
16	1136	TS119_2	2.08	0.67	-1.41	22.7	22.7	0.1	0.9	0.89	-0.01
17	1138	TS229_4	37.74	0	-37.74	50.3	51.1	0.8	0.76	0.68	-0.08
18	1149	TS097_1	0.25	10.1	9.85	32.6	32.5	-0.1	0	0	0
19	1138	TS185_1	0.13	0.52	0.39	61.5	62.6	1.1	0.67	0.58	-0.09
20	1138	TS185_4	0.82	1.08	0.26	57.9	59.3	1.3	0.67	0.61	-0.06
21	1128	TS110_1	48.57	1.45	-47.12	48.4	48.9	0.5	0.88	0.86	-0.02
22	1126	TS470_1	347.18	12.66	-334.52	36.5	37.3	0.8	0.58	0.55	-0.03
23	1126	TS470_2	351.33	13.52	-337.81	36.5	37.1	0.5	0.5	0.47	-0.03
24	1108	TS119_5	4.07	n/a	n/a	16.1	n/a	n/a	0.78	n/a	n/a
25	1126	TS035_1	1.74	n/a	n/a	43.7	n/a	n/a	0.44	n/a	n/a

Continued on next page

Table S3 – continued from previous page

No	Target	Model	Clashscore			RMSD			INF		
			Before	After	Δ	Before	After	Δ	Before	After	Δ
26	1126	TS076_2	36.42	3.35	-33.07	49.3	50	0.7	0.75	0.73	-0.02
27	1128	TS239_3	54.2	6.72	-47.48	24	24.8	0.7	0.78	0.69	-0.09
28	1149	TS177_1	361.22	31.53	-329.69	29	29.1	0.1	0	0	0
29	1136	TS029_2	2.5	n/a	n/a	56.4	n/a	n/a	0.44	n/a	n/a
30	1136	TS392_1	80.33	n/a	n/a	41.3	n/a	n/a	0.82	n/a	n/a
31	1138	TS035_4	0.61	0.78	0.17	81.2	81.6	0.4	0.77	0.75	-0.02
32	1126	TS110_1	85.5	1.2	-84.3	45.5	46	0.5	0.78	0.79	0.01
33	1126	TS110_2	6.01	0.69	-5.32	46.8	47.3	0.5	0.77	0.77	0
34	1126	TS110_3	19.62	1.2	-18.42	45.9	46.2	0.3	0.75	0.75	0
35	1128	TS416_4	4.08	1.71	-2.37	32.9	32.9	0	0.89	0.88	-0.01
36	1136	TS110_1	89.66	2.42	-87.24	50.4	50.8	0.4	0.83	0.8	-0.03
37	1107	TS392_1	19.92	15.41	-4.51	16.7	16.4	-0.3	0.78	0.78	0
38	1107	TS392_2	9.51	9.52	0.01	18	17.9	-0.2	0.74	0.75	0.01
39	1107	TS392_3	4.98	11.78	6.8	18.2	18.1	-0.1	0.72	0.72	0
40	1107	TS392_4	4.07	18.11	14.04	17.9	17.7	-0.2	0.76	0.72	-0.04
41	1107	TS392_5	9.95	9.52	-0.43	19.6	19.6	-0.1	0.73	0.77	0.04
42	1156	TS177_1	302.02	n/a	n/a	23.1	n/a	n/a	0	n/a	n/a
43	1116	TS248_3	10.93	3.78	-7.15	28.6	28.8	0.2	0.63	0.63	0
44	1126	TS185_1	0.09	1.8	1.71	33.5	33.9	0.4	0.7	0.66	-0.04
45	1126	TS185_2	0.09	2.06	1.97	33.6	34.2	0.5	0.71	0.69	-0.02
46	1136	TS470_5	355.56	n/a	n/a	40.9	n/a	n/a	0.54	n/a	n/a
47	1128	TS470_3	112.49	2.37	-110.12	32.7	33.1	0.4	0.84	0.85	0.01
48	1138	TS076_4	5.72	1.52	-4.2	58.2	59.3	1.1	0.8	0.72	-0.08
49	1126	TS054_1	2.06	n/a	n/a	41.5	n/a	n/a	0.83	n/a	n/a
50	1136	TS147_3	0.25	n/a	n/a	43.2	n/a	n/a	0.87	n/a	n/a
51	1138	TS076_3	1.3	0.87	-0.43	40.9	42.2	1.3	0.81	0.71	-0.1
52	1156	TS054_5	6.47	n/a	n/a	25.1	n/a	n/a	0	n/a	n/a
53	1126	TS177_1	254.25	n/a	n/a	37.9	n/a	n/a	0.36	n/a	n/a
54	1128	TS285_3	1.84	1.05	-0.79	37.9	38.3	0.4	0.82	0.84	0.02

Continued on next page

Table S3 – continued from previous page

No	Target	Model	Clashscore			RMSD			INF		
			Before	After	Δ	Before	After	Δ	Before	After	Δ
55	1126	TS470_4	393.19	12.41	-380.78	36.8	37.4	0.6	0.56	0.53	-0.03
56	1136	TS110_3	58.28	2	-56.28	52.4	53	0.5	0.81	0.79	-0.02
57	1116	TS470_5	116.76	7.36	-109.4	17.6	17.3	-0.3	0.79	0.76	-0.03
58	1107	TS119_2	2.26	n/a	n/a	19.7	n/a	n/a	0.71	n/a	n/a
59	1107	TS128_2	110.51	n/a	n/a	17.6	n/a	n/a	0.76	n/a	n/a
60	1108	TS128_2	108.9	n/a	n/a	17.6	n/a	n/a	0.79	n/a	n/a
61	1136	TS128_5	127.46	2.17	-125.29	25	25.2	0.2	0.85	0.84	-0.01
62	1136	TS239_3	30.65	3.33	-27.32	56.1	56.6	0.5	0.76	0.74	-0.02
63	1126	TS147_5	0	n/a	n/a	49.9	n/a	n/a	0.84	n/a	n/a
64	1107	TS163_1	19.45	15.86	-3.59	14.3	14.3	0	0.61	0.59	-0.02
65	1136	TS470_2	373.77	18.61	-355.16	41.4	41.6	0.3	0.56	0.51	-0.05
66	1126	TS185_4	0.17	1.98	1.81	33.6	33.9	0.3	0.65	0.62	-0.03
67	1126	TS128_2	12.2	n/a	n/a	43.2	n/a	n/a	0.84	n/a	n/a
68	1126	TS416_5	38.07	2.84	-35.23	51.5	52	0.5	0.78	0.78	0
69	1156	TS235_5	138.41	n/a	n/a	20.2	n/a	n/a	0	n/a	n/a
70	1126	TS239_5	119.37	1.37	-118	46.4	46.8	0.4	0.76	0.76	0
71	1116	TS470_3	103.59	5.97	-97.62	17.7	18	0.3	0.78	0.76	-0.02
72	1116	TS035_3	10.93	n/a	n/a	24	n/a	n/a	0.73	n/a	n/a
73	1116	TS035_4	15.69	n/a	n/a	23.6	n/a	n/a	0.74	n/a	n/a
74	1116	TS035_5	11.52	n/a	n/a	25.4	n/a	n/a	0.7	n/a	n/a
75	1116	TS285_4	1.39	n/a	n/a	16.4	n/a	n/a	0.67	n/a	n/a
76	1136	TS128_4	69.55	1.92	-67.63	24.7	24.8	0.2	0.86	0.85	-0.01
77	1126	TS470_5	416.69	14.72	-401.97	36.3	36.7	0.4	0.42	0.4	-0.02
78	1128	TS470_5	105.79	3.55	-102.24	32.5	32.6	0.2	0.83	0.82	-0.01
79	1128	TS392_3	90.97	2.37	-88.6	49.2	49.6	0.4	0.63	0.65	0.02
80	1126	TS185_5	0.77	2.06	1.29	33.6	33.8	0.2	0.66	0.62	-0.04
81	1138	TS054_3	3.55	n/a	n/a	39.9	n/a	n/a	0.84	n/a	n/a
82	1138	TS076_2	3.03	0.91	-2.12	40.9	41.7	0.8	0.84	0.76	-0.08
83	1128	TS238_2	313.07	20.73	-292.34	26.6	27.3	0.7	0.79	0.77	-0.02

Continued on next page

Table S3 – continued from previous page

No	Target	Model	Clashscore			RMSD			INF		
			Before	After	Δ	Before	After	Δ	Before	After	Δ
84	1116	TS470_1	117.79	6.56	-111.23	17.4	17.2	-0.2	0.81	0.78	-0.03
85	1136	TS110_4	18.35	2.25	-16.1	44.4	44.6	0.3	0.82	0.81	-0.01
86	1126	TS444_1	351.93	12.31	-339.62	37.5	38.2	0.7	0.5	0.45	-0.05
87	1136	TS177_1	236.44	5.85	-230.59	71.6	73.3	1.7	0.44	0.47	0.03
88	1136	TS110_2	43.3	1.75	-41.55	47.5	47.6	0.2	0.82	0.8	-0.02
89	1138	TS239_5	89.03	4.81	-84.22	49.7	51.5	1.7	0.7	0.59	-0.11
