# Supplementary material for: Unknotting RNA: A method to resolve computational artifacts
Source: PLoS Comput Biol. 2025 Mar 20;21(3):e1012843. doi: 10.1371/journal.pcbi.1012843 (PMC11925458; doi:10.1371/journal.pcbi.1012843)
Supplement: S4 Table — Δ represents the difference between the post- and pre-disentanglement measurements (Δ = After – Before). n/a indicates that the protocol failed for the given model, so post-disentanglement and Δ values are not available. (PDF) [file pcbi.1012843.s004.pdf]

**Table S4:** Evaluation of RNA-Puzzles predictions before and after the application of the disentanglement protocol. Δ represents the difference between the post- and pre-disentanglement measurements (Δ = After – Before). *n/a* indicates that the protocol failed for the given model, so post-disentanglement and Δ values are not available.

| No | Target | Model | Clashscore | | | RMSD | | | INF | | |
|---|---|---|---|---|---|---|---|---|---|---|---|
| | | | Before | After | Δ | Before | After | Δ | Before | After | Δ |
| 1 | PZ16b | 3dRNAAS2_3 | 32.81 | 32.92 | 0.11 | 6 | 6 | 0 | 0.78 | 0.73 | -0.05 |
| 2 | PZ18 | Chen_1 | 0.43 | n/a | n/a | 3.7 | n/a | n/a | 0.83 | n/a | n/a |
| 3 | PZ18 | Chen_2 | 2.17 | 19.69 | 17.52 | 6.6 | 7.5 | 0.9 | 0.84 | 0.72 | -0.12 |
| 4 | PZ18 | Das_1 | 13.05 | n/a | n/a | 5.5 | n/a | n/a | 0.9 | n/a | n/a |
| 5 | PZ18 | Das_2 | 16.09 | n/a | n/a | 3.9 | n/a | n/a | 0.9 | n/a | n/a |
| 6 | PZ18 | Das_3 | 9.57 | n/a | n/a | 5.6 | n/a | n/a | 0.91 | n/a | n/a |
| 7 | PZ18 | Das_4 | 13.05 | 19.62 | 6.57 | 5.7 | 6.4 | 0.7 | 0.87 | 0.81 | -0.06 |
| 8 | PZ18 | DasORIGINAL_2 | 8.7 | 18.33 | 9.63 | 4 | 5.3 | 1.3 | 0.91 | 0.85 | -0.06 |
| 9 | PZ18 | DasORIGINAL_3 | 9.13 | 21.4 | 12.27 | 3.2 | 4.2 | 1 | 0.91 | 0.85 | -0.06 |
| 10 | PZ18 | Dokholyan_1 | 8.7 | 6.54 | -2.16 | 8.4 | 8.7 | 0.3 | 0.72 | 0.68 | -0.04 |
| 11 | PZ18 | Dokholyan_2 | 14.35 | n/a | n/a | 12 | n/a | n/a | 0.74 | n/a | n/a |
| 12 | PZ18 | RNAComposer_5 | 13.48 | n/a | n/a | 15.2 | n/a | n/a | 0.68 | n/a | n/a |
| 13 | PZ18 | solution_0 | 3.48 | n/a | n/a | 0 | n/a | n/a | 1 | n/a | n/a |
| 4 | PZ24 | DasTFN_2 | 5.27 | n/a | n/a | 13.3 | n/a | n/a | 0.85 | n/a | n/a |
| 15 | PZ24 | DasTFN_4 | 1.66 | n/a | n/a | 21.9 | n/a | n/a | 0.86 | n/a | n/a |
| 16 | PZ24 | DasTFN_5 | 4.44 | n/a | n/a | 19.6 | n/a | n/a | 0.87 | n/a | n/a |
| 17 | PZ24 | FARFAR2_1 | 1.66 | 5.6 | 3.94 | 16.8 | 16.7 | -0.1 | 0.86 | 0.83 | -0.03 |
| 18 | PZ24 | FARFAR2_2 | 0.55 | 3.92 | 3.37 | 16.9 | 17 | 0.1 | 0.84 | 0.83 | -0.01 |
| 19 | PZ24 | FARFAR2_3 | 0.83 | 0.56 | -0.27 | 19.1 | 19.6 | 0.5 | 0.87 | 0.88 | 0.01 |
| 20 | PZ24 | FARFAR2_5 | 0.83 | n/a | n/a | 18.4 | n/a | n/a | 0.85 | n/a | n/a |
| 21 | PZ24 | iFoldRNA_4 | 1.94 | n/a | n/a | 27.2 | n/a | n/a | 0.72 | n/a | n/a |
| 22 | PZ24 | Kollmann_3 | 123.16 | 11.24 | -111.92 | 33 | 33.5 | 0.5 | 0.62 | 0.59 | -0.03 |
| 23 | PZ24 | Kollmann_6 | 92.95 | 11.78 | -81.17 | 30.6 | 30.9 | 0.3 | 0.62 | 0.61 | -0.01 |
| 24 | PZ24 | Kollmann_8 | 112.13 | 10.12 | -102.01 | 32.7 | 33 | 0.3 | 0.58 | 0.57 | -0.01 |
| 25 | PZ24 | Vfold3D_3 | 2.22 | 5.04 | 2.82 | 15.5 | 15.1 | -0.4 | 0.84 | 0.84 | 0 |

**Continued on next page**

**Table S4 – continued from previous page**

| No | Target | Model | Clashscore | | | RMSD | | | INF | | |
|---|---|---|---|---|---|---|---|---|---|---|---|
| | | | Before | After | Δ | Before | After | Δ | Before | After | Δ |
| 26 | PZ25 | Das_2 | 123.81 | 26.27 | -97.54 | 7 | 7.2 | 0.2 | 0.73 | 0.72 | -0.01 |
| 27 | PZ25 | Ding_1 | 9.03 | n/a | n/a | 10.6 | n/a | n/a | 0.74 | n/a | n/a |
| 28 | PZ25 | Ding_2 | 12.64 | 21.75 | 9.11 | 8.6 | 8.7 | 0.1 | 0.7 | 0.72 | 0.02 |
| 29 | PZ25 | Ding_3 | 11.29 | n/a | n/a | 9.8 | n/a | n/a | 0.76 | n/a | n/a |
| 30 | PZ25 | SimRNA_3 | 131.89 | 38.48 | -93.41 | 9.6 | 9.5 | -0.1 | 0.75 | 0.65 | -0.1 |
| 31 | PZ26 | Chen_9 | 1.98 | 6.44 | 4.46 | 18.4 | 18.5 | 0.1 | 0.85 | 0.8 | -0.05 |
| 32 | PZ26 | Das_3 | 2.64 | 2.62 | -0.02 | 15.7 | 15.9 | 0.2 | 0.84 | 0.85 | 0.01 |
| 33 | PZ26 | FARFAR2_5 | 1.32 | n/a | n/a | 15 | n/a | n/a | 0.86 | n/a | n/a |
| 34 | PZ26 | TFN_3 | 3.74 | 5.73 | 1.99 | 12.5 | 18.4 | 5.9 | 0.87 | 0.82 | -0.05 |
| 35 | PZ26 | TFN_6 | 5.94 | 1.43 | -4.51 | 17.2 | 15.8 | -1.4 | 0.86 | 0.84 | -0.02 |
| 36 | PZ26tBox | Chen_9 | 1.98 | 4.53 | 2.55 | 18.4 | 14.9 | -3.5 | 0.85 | 0.84 | -0.01 |
| 37 | PZ26tBox | Das_3 | 2.86 | n/a | n/a | 15.7 | n/a | n/a | 0.84 | n/a | n/a |
| 38 | PZ26tBox | FARFAR2_5 | 1.32 | n/a | n/a | 15 | n/a | n/a | 0.86 | n/a | n/a |
| 39 | PZ26tBox | TFN_3 | 3.52 | n/a | n/a | 12.5 | n/a | n/a | 0.87 | n/a | n/a |
| 40 | PZ26tBox | TFN_6 | 6.83 | n/a | n/a | 17.2 | n/a | n/a | 0.86 | n/a | n/a |
| 41 | PZ27 | Das_2 | 7.61 | n/a | n/a | 10.2 | n/a | n/a | 0.86 | n/a | n/a |
| 42 | PZ27 | Das_6 | 6.34 | n/a | n/a | 11.9 | n/a | n/a | 0.86 | n/a | n/a |
| 43 | PZ27 | FARFAR2_6 | 1.32 | 3.1 | 1.78 | 10.5 | 10.7 | 0.2 | 0.85 | 0.84 | -0.01 |
| 44 | PZ27 | RNAComposer_2 | 11.66 | 4.58 | -7.08 | 21.7 | 21.9 | 0.2 | 0.81 | 0.81 | 0 |
| 45 | PZ27 | SimRNA_3 | 139.14 | 6.47 | -132.67 | 61.2 | 61.6 | 0.4 | 0.55 | 0.54 | -0.01 |
| 46 | PZ27tBox | Das_2 | 7.48 | n/a | n/a | 10.2 | n/a | n/a | 0.86 | n/a | n/a |
| 47 | PZ27tBox | Das_6 | 6.59 | n/a | n/a | 11.9 | n/a | n/a | 0.86 | n/a | n/a |
| 48 | PZ27tBox | FARFAR2_6 | 7.1 | 3.77 | -3.33 | 10.5 | 10.7 | 0.2 | 0.85 | 0.85 | 0 |
| 49 | PZ27tBox | RNAComposer_2 | 11.66 | n/a | n/a | 21.7 | n/a | n/a | 0.82 | n/a | n/a |
| 50 | PZ27tBox | SimRNA_3 | 139.14 | 4.99 | -134.15 | 61.2 | 62.2 | 1 | 0.55 | 0.55 | 0 |
| 51 | PZ28 | Bujnicki_4 | 18.74 | 3.54 | -15.2 | 20.1 | 20 | -0.1 | 0.8 | 0.8 | 0 |
| 52 | PZ28 | Bujnicki_5 | 35.71 | 6.01 | -29.7 | 28.3 | 28.7 | 0.4 | 0.8 | 0.79 | -0.01 |
| 53 | PZ28 | SimRNA_5 | 144.93 | n/a | n/a | 29.1 | n/a | n/a | 0.71 | n/a | n/a |
| 54 | PZ28tBox | Bujnicki_4 | 18.56 | 3.18 | -15.38 | 20.1 | 20 | -0.1 | 0.8 | 0.78 | -0.02 |

**Continued on next page**

**Table S4 – continued from previous page**

| No | Target | Model | Clashscore | | | RMSD | | | INF | | |
|---|---|---|---|---|---|---|---|---|---|---|---|
| | | | Before | After | Δ | Before | After | Δ | Before | After | Δ |
| 55 | PZ28tBox | Bujnicki_5 | 36.77 | 4.07 | -32.7 | 28.3 | 28.7 | 0.4 | 0.8 | 0.78 | -0.02 |
| 56 | PZ28tBox | SimRNA_5 | 142.28 | n/a | n/a | 29.1 | n/a | n/a | 0.71 | n/a | n/a |
| 57 | PZ31 | Dfold_3 | 160.26 | 27.48 | -132.78 | 22.8 | 22.9 | 0.1 | 0.56 | 0.52 | -0.04 |
| 58 | PZ31 | Dfold_4 | 154.12 | n/a | n/a | 22.6 | n/a | n/a | 0.45 | n/a | n/a |
| 59 | PZ32 | Boniecki_1 | 127.54 | n/a | n/a | 18.9 | n/a | n/a | 0.48 | n/a | n/a |
| 60 | PZ32 | Boniecki_2 | 160.23 | 20.2 | -140.03 | 18.2 | 18.5 | 0.3 | 0.47 | 0.42 | -0.05 |
| 61 | PZ32 | Boniecki_3 | 113.29 | n/a | n/a | 17 | n/a | n/a | 0.46 | n/a | n/a |
| 62 | PZ32 | Boniecki_4 | 119.85 | n/a | n/a | 16.4 | n/a | n/a | 0.42 | n/a | n/a |
| 63 | PZ32 | Bujnicki_1 | 9.51 | n/a | n/a | 19 | n/a | n/a | 0.55 | n/a | n/a |
| 64 | PZ32 | Chen_5 | 0 | n/a | n/a | 19.1 | n/a | n/a | 0.53 | n/a | n/a |
| 65 | PZ32 | Dfold_3 | 164.77 | n/a | n/a | 13.7 | n/a | n/a | 0.6 | n/a | n/a |
| 66 | PZ32 | Dfold_6 | 137.69 | n/a | n/a | 17.4 | n/a | n/a | 0.46 | n/a | n/a |
| 67 | PZ32 | Dfold_10 | 138.32 | n/a | n/a | 14.8 | n/a | n/a | 0.51 | n/a | n/a |
| 68 | PZ32 | FARFAR2_3 | 1.27 | n/a | n/a | 18.2 | n/a | n/a | 0.58 | n/a | n/a |
| 69 | PZ32 | FARFAR2_5 | 2.54 | n/a | n/a | 20.3 | n/a | n/a | 0.59 | n/a | n/a |
| 70 | PZ32 | FARFAR2_6 | 1.9 | n/a | n/a | 20.2 | n/a | n/a | 0.55 | n/a | n/a |
| 71 | PZ33 | Dfold_3 | 164.13 | n/a | n/a | 13.7 | n/a | n/a | 0.6 | n/a | n/a |
| 72 | PZ33 | Dfold_6 | 138.96 | n/a | n/a | 17.4 | n/a | n/a | 0.46 | n/a | n/a |
| 73 | PZ33 | Dfold_10 | 140.23 | n/a | n/a | 14.8 | n/a | n/a | 0.51 | n/a | n/a |
| 74 | PZ34 | Chen_7 | 0 | 16.24 | 16.24 | 17.4 | 17.7 | 0.3 | 0.68 | 0.64 | -0.04 |
| 75 | PZ34 | Chen_9 | 0 | 25.57 | 25.57 | 18.9 | 19.1 | 0.2 | 0.7 | 0.7 | 0 |
| 76 | PZ34 | Nithin_4 | 0 | 19.54 | 19.54 | 17.4 | 17.3 | -0.1 | 0.63 | 0.62 | -0.01 |
| 77 | PZ34 | SimRNA_1 | 138.21 | 30.8 | -107.41 | 14.2 | 14.5 | 0.3 | 0.76 | 0.67 | -0.09 |
| 78 | PZ35 | Bujnicki_2 | 106.8 | n/a | n/a | 33.6 | n/a | n/a | 0.71 | n/a | n/a |
| 79 | PZ35 | Bujnicki_5 | 103.64 | n/a | n/a | 21 | n/a | n/a | 0.71 | n/a | n/a |
| 80 | PZ35 | RNAComposer_2 | 24.42 | n/a | n/a | 30.8 | n/a | n/a | 0.65 | n/a | n/a |
| 81 | PZ35 | RNAComposer_3 | 15.61 | n/a | n/a | 25.7 | n/a | n/a | 0.68 | n/a | n/a |
| 82 | PZ35 | RNAComposer_4 | 12.44 | n/a | n/a | 26.6 | n/a | n/a | 0.69 | n/a | n/a |
| 83 | PZ35 | RNAComposer_5 | 17.41 | n/a | n/a | 27.6 | n/a | n/a | 0.69 | n/a | n/a |

**Continued on next page**

**Table S4 – continued from previous page**

| No | Target | Model | Clashscore | | | RMSD | | | INF | | |
|---|---|---|---|---|---|---|---|---|---|---|---|
| | | | Before | After | Δ | Before | After | Δ | Before | After | Δ |
| 84 | PZ35 | TSR04_2 | 106.8 | 16.09 | -90.71 | 33.6 | 33.8 | 0.2 | 0.71 | 0.73 | 0.02 |
| 85 | PZ35 | TSR04_5 | 103.64 | 14.74 | -88.9 | 21 | 21.2 | 0.2 | 0.71 | 0.67 | -0.04 |
| 86 | PZ35 | TSR07_2 | 24.42 | n/a | n/a | 30.8 | n/a | n/a | 0.65 | n/a | n/a |
| 87 | PZ35 | TSR07_3 | 15.61 | n/a | n/a | 25.7 | n/a | n/a | 0.68 | n/a | n/a |
| 88 | PZ35 | TSR07_4 | 12.44 | n/a | n/a | 26.6 | n/a | n/a | 0.69 | n/a | n/a |
| 89 | PZ35 | TSR07_5 | 17.41 | n/a | n/a | 27.6 | n/a | n/a | 0.69 | n/a | n/a |
| 90 | PZ36 | Bujnicki_5 | 103.78 | 16.74 | -87.04 | 21.6 | 21.9 | 0.3 | 0.72 | 0.67 | -0.05 |
| 91 | PZ36 | Nithin_5 | 80.88 | 7.92 | -72.96 | 31 | 31.3 | 0.3 | 0.71 | 0.72 | 0.01 |
| 92 | PZ36 | RNAComposer_3 | 12.43 | n/a | n/a | 26.3 | n/a | n/a | 0.76 | n/a | n/a |
| 93 | PZ36 | RNAComposer_4 | 9.04 | n/a | n/a | 28.7 | n/a | n/a | 0.74 | n/a | n/a |
| 94 | PZ36 | RNAComposer_5 | 11.76 | 5.66 | -6.1 | 29 | 29.1 | 0.1 | 0.74 | 0.72 | -0.02 |
| 95 | PZ37 | DasARESapo_2 | 22.91 | 19.9 | -3.01 | 11.9 | 12.4 | 0.5 | 0.66 | 0.62 | -0.04 |
| 96 | PZ37 | DasARESapo_3 | 17.83 | n/a | n/a | 15.6 | n/a | n/a | 0.69 | n/a | n/a |
| 97 | PZ37 | DasFARFAR2apo_3 | 18.33 | n/a | n/a | 15.4 | n/a | n/a | 0.68 | n/a | n/a |
| 98 | PZ37 | DasFARFAR2ARESholo_1 | 15.27 | n/a | n/a | 12.9 | n/a | n/a | 0.64 | n/a | n/a |
| 99 | PZ37 | DasSWMap_1 | 18.33 | 26.54 | 8.21 | 13.9 | 13.8 | -0.1 | 0.67 | 0.65 | -0.02 |
| 100 | PZ37 | Ding_1 | 9.16 | 18.39 | 9.23 | 21.4 | 21.4 | 0 | 0.39 | 0.36 | -0.03 |
| 101 | PZ37 | Yang_2 | 7.64 | n/a | n/a | 10.4 | n/a | n/a | 0.67 | n/a | n/a |
| 102 | PZ37 | Yang_10 | 24.95 | n/a | n/a | 23.2 | n/a | n/a | 0.32 | n/a | n/a |
| 103 | PZ39 | Dfold_4 | 116.32 | n/a | n/a | 18.2 | n/a | n/a | 0.57 | n/a | n/a |
| 104 | PZ39 | Perez_7 | 0.69 | 15.45 | 14.76 | 23.9 | 24 | 0.1 | 0.87 | 0.82 | -0.05 |
| 105 | PZ39 | Perez_9 | 0.69 | n/a | n/a | 11.9 | n/a | n/a | 0.7 | n/a | n/a |
| 106 | PZ39 | Xiao_1 | 4.17 | 10.91 | 6.74 | 23.9 | 24 | 0.1 | 0.8 | 0.8 | 0 |
