# Supplementary material for: Unknotting RNA: A method to resolve computational artifacts
Source: PLoS Comput Biol. 2025 Mar 20;21(3):e1012843. doi: 10.1371/journal.pcbi.1012843 (PMC11925458; doi:10.1371/journal.pcbi.1012843)
Supplement: S1 Text — (PDF) [file pcbi.1012843.s005.pdf]

Unknotting RNA: a method to resolve computational artifacts
Supporting Figures and Appendices

Simón Poblete[1,2*], Mikolaj Mlynarczyk[3], Marta Szachniuk[3,4*]

1 Facultad de Ingeniería, Arquitectura y Diseño, Universidad San Sebastián, Santiago, Chile
2 Centro BASAL Ciencia & Vida, Universidad San Sebastián, Santiago, Chile
3 Institute of Computing Science, Poznan University of Technology, Poznan, Poland
4 Institute of Bioorganic Chemistry, Polish Academy of Sciences, Poznan, Poland

* spoblete@cienciavida.org (SP); mszachniuk@cs.put.poznan.pl (MS)

Contents

Fig A 2
Fig B 3
Fig C 4
Fig D 5
Fig E 6
Fig F 7
Fig G 8
Fig H 9
Fig I 10
Fig J 11
Appendix A 12
Appendix B 15

References **16**

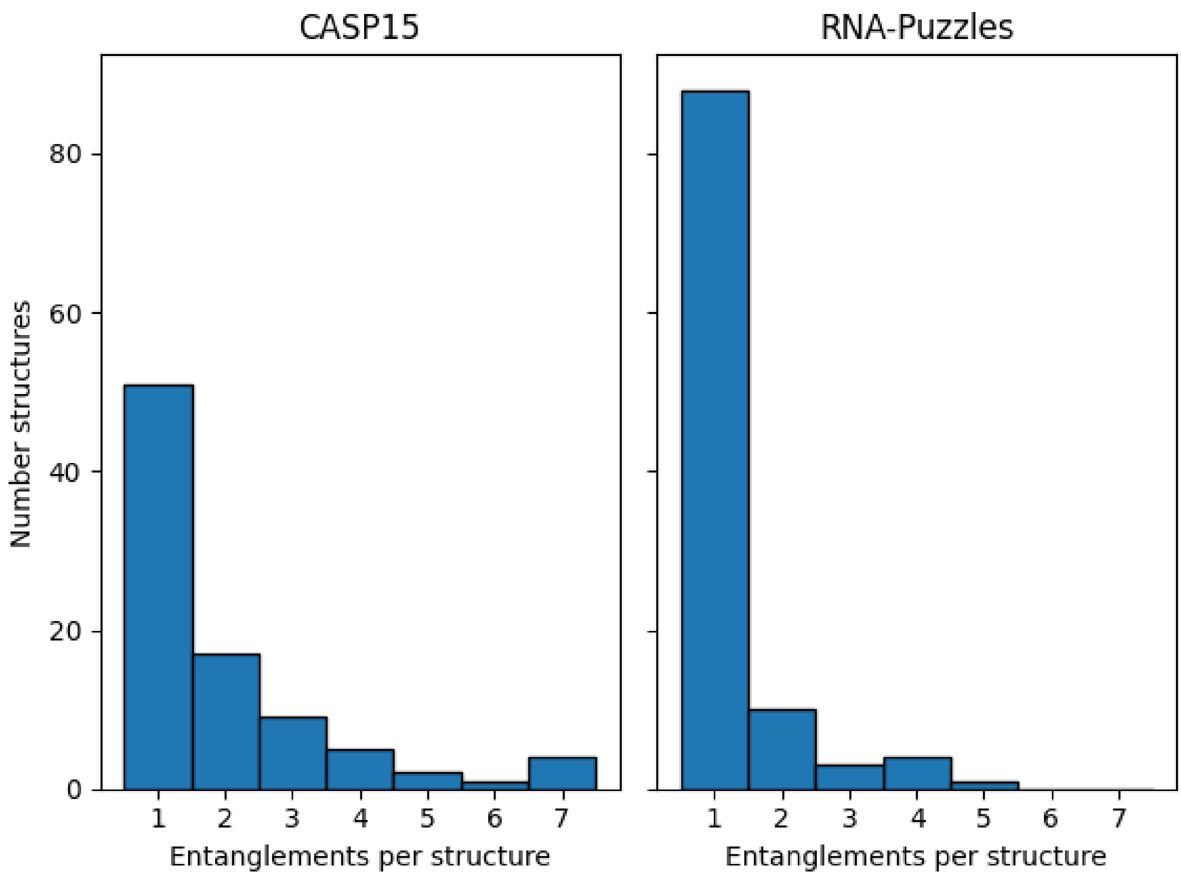

Figure A: Number of entanglements per structure in the CASP15 (omitting R1138TS239_5 with 15 entanglements) and RNA-Puzzles datasets.

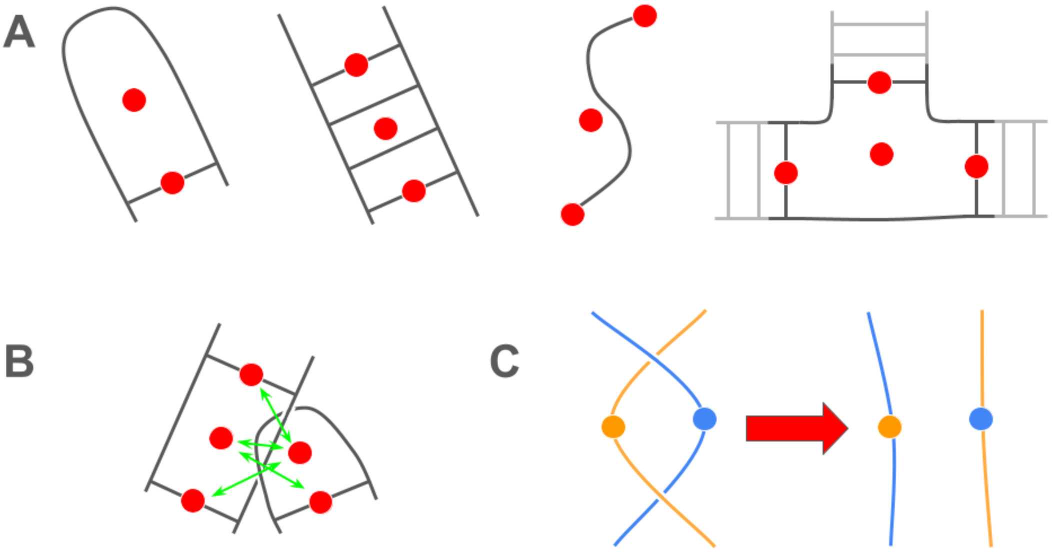

Figure B: (A) Virtual sites defined for a hairpin, stem, single strand, and junction. (B) Repulsive energies drive the nucleotides toward an untangled conformation. (C) For short single strands, specific virtual sites function as attractive and repulsive forces, leading to disentanglement.

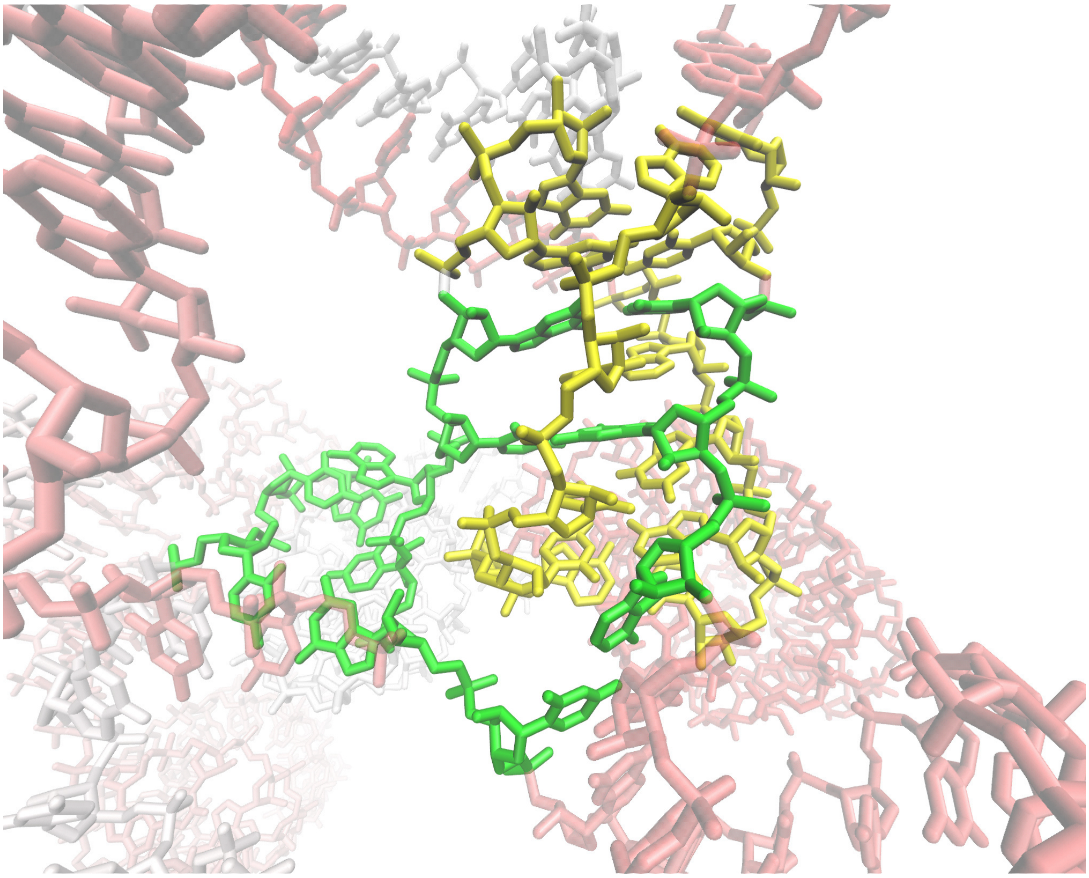

Figure C: The RNA 3D model exhibiting five entanglements (R1138TS076_2). The model contains six loops that can be merged into two large domains, represented in yellow and green. One of the entanglements is of type D&D, which is the only instance of this type not resolved by our protocol across the entire benchmark set.

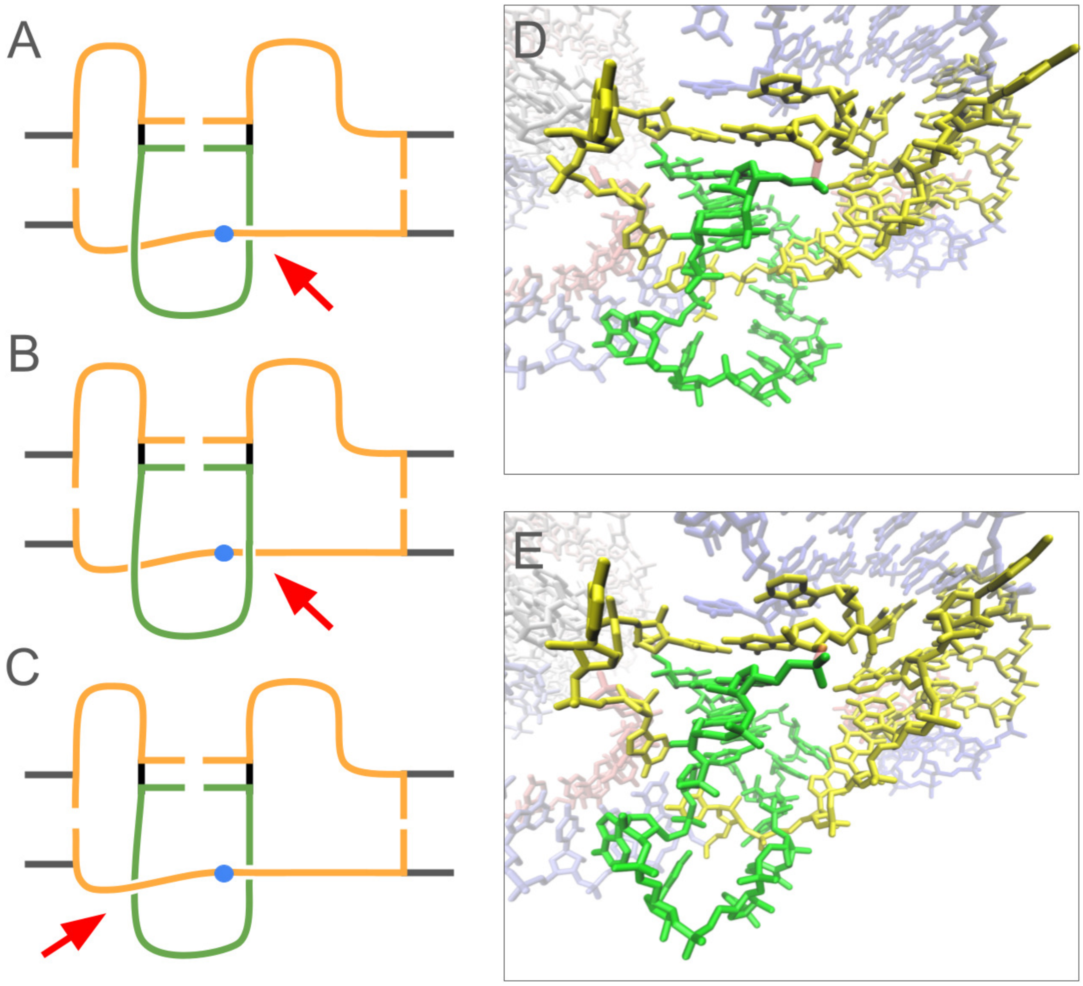

Figure D: Transformation of the L&L interlace to an L(L) lasso, exemplified using the R1116TS035_3 model. The original structure (A) is corrected by exchanging loop positions near the intersection point (blue bubble) as shown in (B). As a result, the yellow loop encloses the green loop in a 3D space. If the correction is applied farther from the intersection point, as in (C), the entanglement can be resolved. The 3D structures before and after the correction are displayed in (D) and (E).

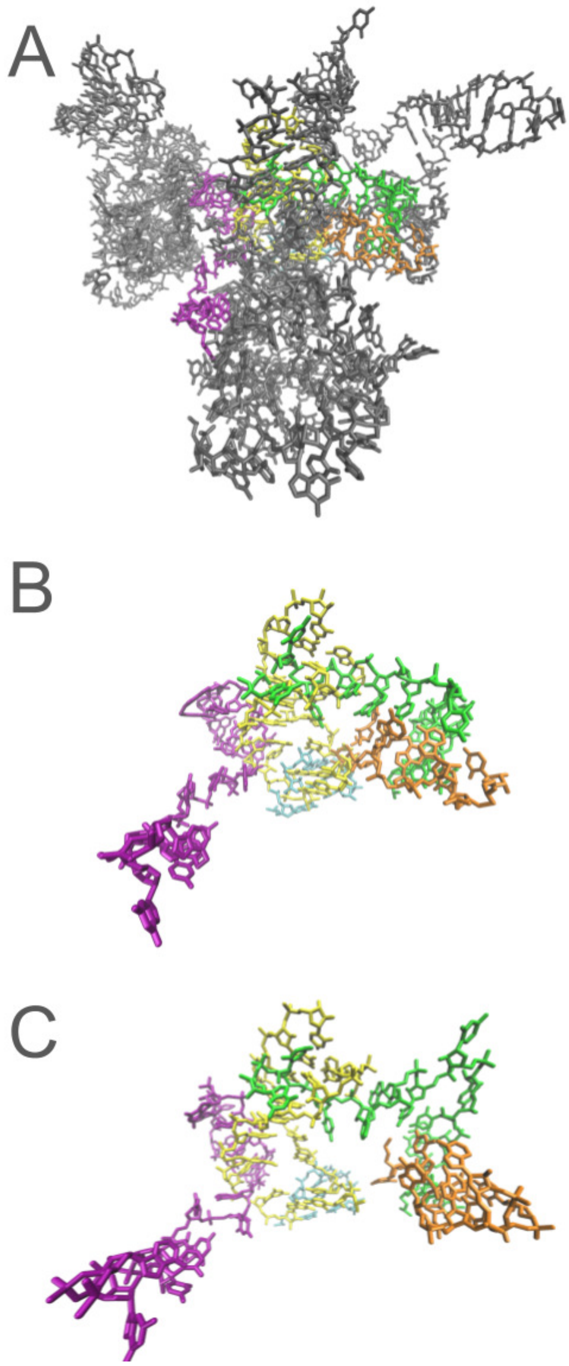

Figure E: The R1126TS444_1 model from CASP15 with a D(L) lasso – the only one of its type not resolved by the protocol. In (A), loops involved in entanglements (buried within the structure) are colored, while the rest of the structure is shown in gray. The entangled and disentangled conformations, shown in (B) and (C) respectively, illustrate how the protocol removes some entanglements but leaves the D(L) between the yellow and green fragments modified, yet not fully corrected.

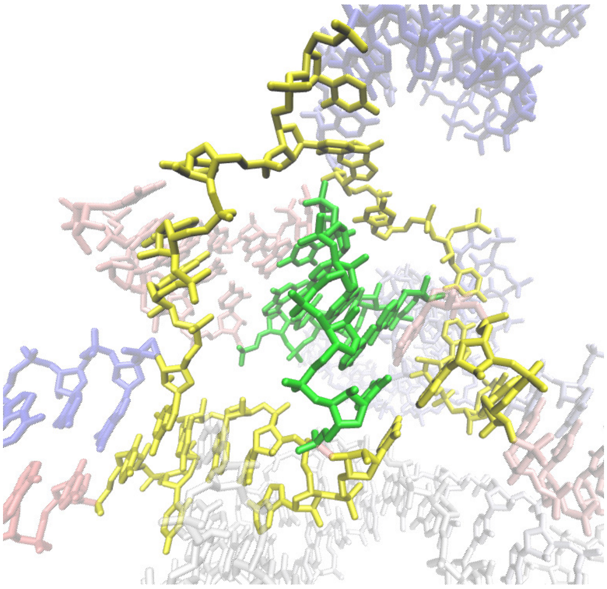

Figure F: A typical case of a large loop enclosing several dinucleotide steps. In this example (the R1128TS392_3 model from CASP15 dataset), four L(D) lassos are practically not affected by the disentanglement protocol.

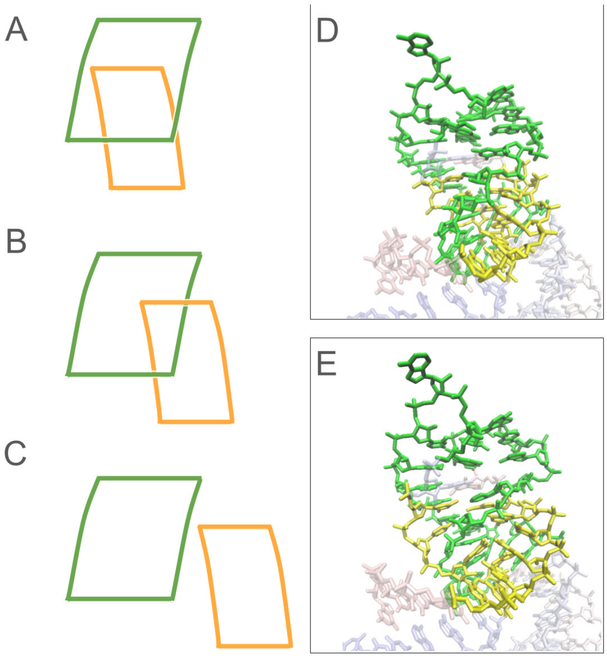

Figure G: Repulsive energy terms separate the loops forming an L(L) lasso (A), guiding the structure through an intermediate conformation (B) towards the disentangled state (C). If the energy terms are too weak or the simulation too short, the lasso can degenerate into an L&L interlace conformation (B), as illustrated in (D) and (E) for the PZ27_SimRNA_3 model from the RNA-Puzzles dataset.

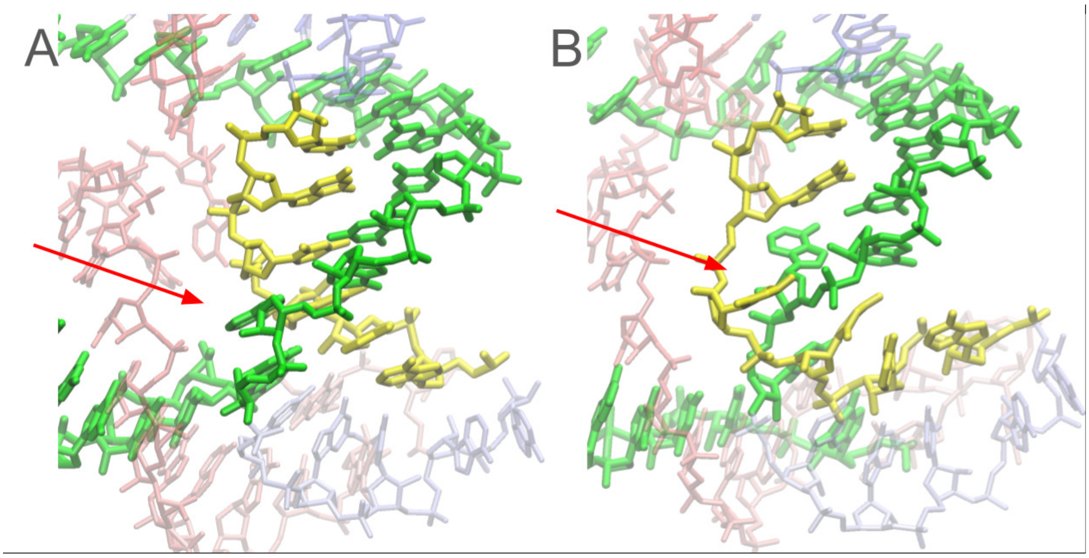

Figure H: The L(S) lasso in the PZ25_SimRNA_3 contains base pairs, shown in (A) which are destroyed by the disentanglement procedure as displayed in (B).

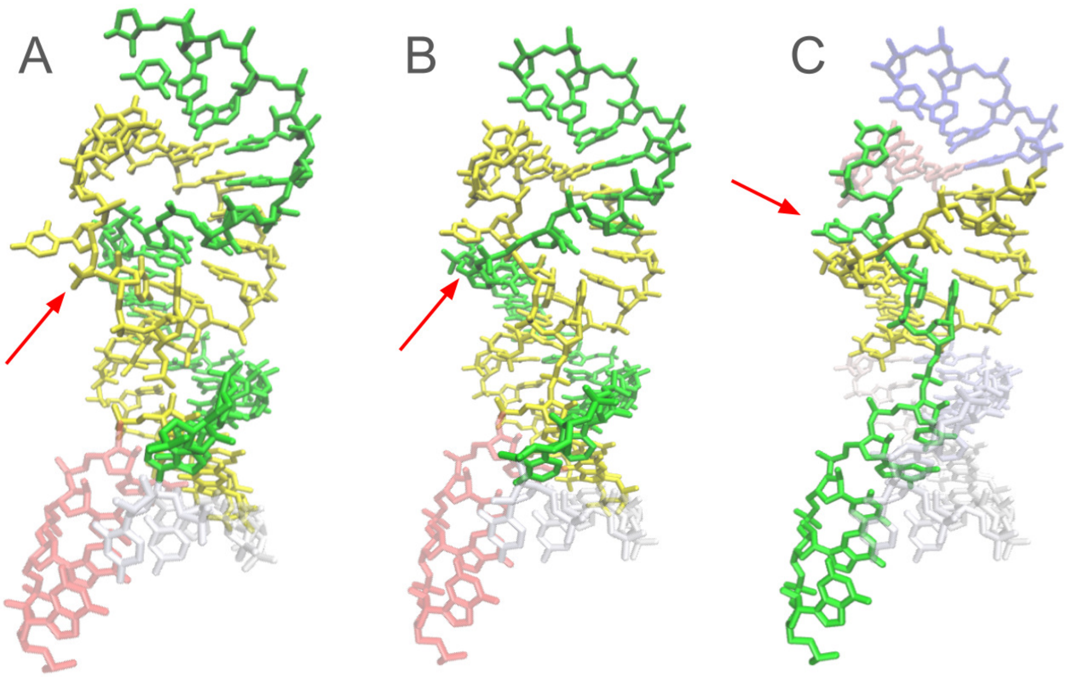

Figure I: The L(S) lasso found in the PZ32_Boniecki_2 model (A) is successfully resolved, resulting in the disentangled structure (B). The original lassoing loop is closed by a pseudoknot. However, the corrected configuration introduces a new L(S) lasso, where the punctured loop is closed by a non-pseudoknotted base pair. A similar situation is observed in the PZ24_FARFAR2_2 model from RNA-Puzzles set.

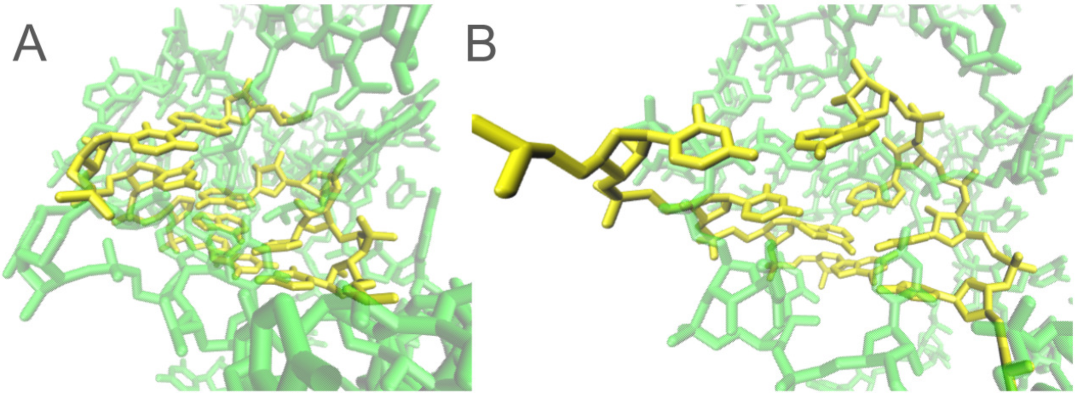

Figure J: Clash removal in the R1128TS392_3 model from CASP15 with a ClashScore of 90.97 (A) results in a structure (B) that is classified as entangled.

Appendix A: Commands to run SPQR disentangling simulations

SPQR can be downloaded by simply cloning its source by running

```
git clone https://github.com/srnas/spqr.git
```

The installation is done by running into the `src/` directory the following commands:

```
autoconf
./configure
make
make install
```

For running a short example of a disentanglement procedure, we can annotate the structure `R1126TS076_2` from the CASP15 database. It contains a single L&L entanglement, which can be obtained from the classification given by RNAspider webserver `https://rnaspider.cs.put.poznan.pl/` , together with its secondary structure. In the `spqr/tools` directory, find the `disentangle_from_RNAspider.py`. We can run

```
python disentangle_from_RNAspider.py -i R1126TS076_2.pdb
                -s R1126TS076_2_report.csv -t R1126TS076_2.ss -o refined
```

where `R1126TS076_2.pdb` is the pdb structure, `R1126TS076_2_report.csv` is the report obtained from the RNAspider webserver, and `R1126TS076_2.ss` is the secondary structure written in three lines : a comment starting with `>`, the sequence and the secondary structure in Vienna notation. `refined` is the name that we will give to the directory where the results will be stored.
The resulting structure is stored in `refined/AA_refined.pdb` in all-atom description, and in `refined/SPQR_refined.pdb` in its coarse-grained representation.
This is a crude backmapped version which is disentangled but might require additional refinement for fixing some broken bonds or small atom clashes. In principle, it can be fixed by a short MD energy minimization, as described below. Still, we can help a bit by running the script `spqr/tools/SPQR_BMAP_RELAX.py` as

```
python SPQR_BMAP_RELAX.py -i AA_refined.pdb -r SPQR_refined.pdb -o AA_better.pdb
```

which reduces the clashes and improves the quality of the structure, specially in the backbone. The detailed parameters of the simulations used in the structures of both datasets are listed below.

1. Preprocessing The script `spider2spqr.py` script is found in the directory `spqr/tools/python3/` . The file containing the definition of the entanglements has to be obtained from the RNAspider webserver (`https://rnaspider.cs.put.poznan.pl`). For obtaining a file with the information of reduced entanglements suitable for SPQR, one must run

   ```
   python spider2spqr.py -i $SPIDERFILE.csv -o $SPQRLIST.lst
                                   -p $PDBFILE.pdb -t $SEC_STRUCT.ss
   ```

   The RNAspider results must be introduced in .csv format. The original pdb file also has to be provided, together with the secondary structure in Vienna format. This file must contain at least three lines: a comment, the sequence, and the dot-bracket structure. The output file corresponds to a parameter file, which must be renamed as `linked_loops.lst` in the directory of a SPQR simulation.

2. SPQR simulations

   - **Energy minimization**
     First, the energy of the original PDB structure with the SPQR energy function is minimized by executing the following script, which is found in the directory `tools/`

     ```
     ./SPQR_REFINE -i $PDBFILE.pdb
     ```

     The resulting structures are stored in the directory `refSPQR_$PDBFILE` under the name `/refSPQR_$PDBFILE.p00.mc`. This specific, binary file will be the initial condition of the untangling simulation. In addition, the script generates a pdb with the same name which is useful for visualization, and the structure `init.pdb` which is the original structure in SPQR format and will be used in the refinement procedure later.
   - **Untangling.**
     Untangling simulations were run with the binary `SPQR_cMC` at temperature of 9 units and 6000 Monte Carlo sweeps, with the parameters `MC_PH_XYZ` = 2, `MC_NT_XYZ` = 2 and `MC_NT_ANGLE` = 0.5, 0.5 . The file `linked_loops.lst` generated previously must be present in the simulation directory.
   - **Energy minimization, after removing entanglement.**
     A more controlled simulation is run by executing using the previous structure as initial condition with the simulated annealing binary `SPQR_wSA` with `MC_STEPS` = 1000 , `MC_PH_XYZ` = 2, `MC_NT_XYZ` = 1 and `MC_NT_ANGLE` = 0.3, 1 . Additionally, `SA_TINI` = 9 , `SA_NT` = 50.
   - **Pull towards original structure.**
     The pulling is performed using the initial structure as template for the ERMSD-restrained simulation, which is stored in the folder of the first energy minimization step, as `refSPQR_$PDBFILE/init.pdb`. We can run

     ```
     python spqr2ermsd.py -i init.pdb -k1 10 -kr 10 -o ermsd_frags.lst
     ```

     to obtain the file `ermsd_frags.lst` which must be included in the simulation folder. The python script is found in the `tools/python3/` directory. , with the binary file `SPQR_cSA`. The parameters are `MC_STEPS` = 100000 , `MC_PH_XYZ` = 2, `MC_NT_XYZ` = 1 and `MC_NT_ANGLE` = 0.3, 1 , `SA_TINI` = 9 , `SA_NT` = 5 . The simulation was run using as a template the
   - **Relaxation of all-atom structures.**
     The relaxation of the backmapped structures was performed using the script `spqr/tools/SPQR_BMAP_RELAX.py`. The position of each phosphorus atom was relaxed if their distance from its neighboring O3' or O5' atoms was larger than 1.7Å. If so, a Monte Carlo procedure of 10000 sweeps at zero temperature was performed on each backbone atom between the nucleotides that enclose the phosphate group. After this, the distance between the phosphorus atom and its neighbors is checked again and the procedure is repeated if needed, with a maximum of 10 times.

3. All-atom simulations

   The force field used for these simulations was AMBER99 [1] with χOL [2] and parmbsc0 [3] corrections.

   - The energy of the structure was minimized by steepest descent algorithm with a tolerance of 1.0 kJ/mol/nm for the maximum force and 10000 steps.

- The pulling simulation was done using the Velocity Rescale thermostat [4] with a time constant of 0.1 ps and a temperature of 10 K for 10000 time steps of 0.002 ps. RMSD and ERMSD (with cutoff 3) moving restrictions were imposed with PLUMED [5] using the original structure as reference, with coupling constants increasing from 0 to 5000000 kJ/mol/nm^2 and 20000 kJ/mol at a time of 20 ps. These values can reach 10000000 kJ/mol/nm^2 and 100000 kJ/mol, respectively, in order to obtain slightly better values of INF and RMSD. In addition, for most systems the pulling simulation can be done for 4000 time steps provided the coupling constants assume large values. The times reported in the main text were measured using 8 threads in an AMD Ryzen Threadripper 2950X 16-Core Processor and a single GPU NVIDIA GeForce RTX 2080 SUPER.

Appendix B: Energy terms of CG simulations

The energy of attraction/repulsion between virtual sites and is given by

$$U_{a,b} = K r_{ab}^2 \tag{1}$$

where a, b stand for the label of the virtual site, which can be a center of mass or a closing loop, r_{ab} for the distance between these points, and K is a constant which is positive for repulsive terms and negative for attractive interactions. The constant has a value of 500 reduced units in SPQR units.

References

1. Cornell, Wendy D and Cieplak, Piotr and Bayly, Christopher I and Gould, Ian R and Merz, Kenneth M and Ferguson, David M and Spellmeyer, David C and Fox, Thomas and Caldwell, James W and Kollman, Peter A A second generation force field for the simulation of proteins, nucleic acids, and organic molecules. J. Am. Chem. Soc. 1995; 117:5179–5197.

2. Banás, Pavel and Hollas, Daniel and Zgarbová, Marie and Jurecka, Petr and Orozco, Modesto and Cheatham III, Thomas E and Sponer, Jirí and Otyepka, Michal. Performance of molecular mechanics force fields for RNA simulations: stability of UUCG and GNRA hairpins. J. Am. Chem. Sic. 2010; 6:3836–3849.

3. Pérez, Alberto and Marchán, Iván and Svozil, Daniel and Sponer, Jiri and Cheatham, Thomas E and Laughton, Charles A and Orozco, Modesto. Refinement of the AMBER force field for nucleic acids: improving the description of α/γ conformers. Biophys. J. 2007; 92:3817–3829.

4. Bussi, Giovanni and Donadio, Davide and Parrinello, Michele. Canonical sampling through velocity rescaling J. Chem. Phys. 2007; 126: 014101.

5. Bonomi, Massimiliano and Branduardi, Davide and Bussi, Giovanni and Camilloni, Carlo and Provasi, Davide and Raiteri, Paolo and Donadio, Davide and Marinelli, Fabrizio and Pietrucci, Fabio and Broglia, Ricardo A and others. PLUMED: A portable plugin for free-energy calculations with molecular dynamics. Comp. Phys. Comm. 2009; 180: 1961–1972.
